# Dual ROS modulation by MnO_2_-integrated collagen hydrogel enhances hiPSC-derived endothelial progenitor cell therapy for critical limb ischemia

**DOI:** 10.7150/thno.127711

**Published:** 2026-04-22

**Authors:** Zhen Zhang, Liang Huang, Gaocheng Gai, Bingbing Xie, Yijing Zhao, Lei Xu, Qiuling Xiang

**Affiliations:** 1 Center for Stem Cell Biology and Tissue Engineering, Key Laboratory for Stem Cells and Tissue Engineering of Ministry of Education, Zhongshan School of Medicine, Sun Yat-sen University, Guangzhou 510080, China.; 2 Department of Physiology, Zhongshan School of Medicine, Sun Yat-sen University, Guangzhou 510080, China.; 3 Shenshan Medical Center, Memorial Hospital of Sun Yat-Sen University.; 4 Guangdong Provincial Key Laboratory of Malignant Tumor Epigenetics and Gene Regulation, Medical Research Center, Sun Yat-sen Memorial Hospital, Sun Yat-sen University, Guangzhou 510120, China.; 5 RNA Biomedical Institute, Sun Yat-sen Memorial Hospital, Sun Yat-sen University, Guangzhou 510120, China.

**Keywords:** MnO_2_-NPs, hybrid hydrogel, hiPSC-EPCs, stem cell therapy, limb ischemia

## Abstract

**Rationale:**

Cell therapy shows significant potential in treating ischemic diseases, such as critical limb ischemia. Endothelial progenitor cells (EPCs) are considered ideal candidates, but their clinical efficacy is often limited due to the scarcity of suitable sources and poor post-transplant survival. Human-induced pluripotent stem cell-derived EPCs (hiPSC-EPCs) offer a scalable and promising alternative. Additionally, injectable hybrid hydrogels can enhance cell retention and eliminate harmful components in the microenvironment, such as reactive oxygen species (ROS). However, conventional biomaterials are insufficient in mitigating intracellular oxidative stress induced by ischemia.

**Methods:**

hiPSC-EPCs were generated by inducing hiPSC with growth factors and small molecules. Manganese dioxide nanoparticles (MnO_2_-NPs) were synthesized by dissolving MnO_2_ in an aqueous NaOH solution and neutralizing the mixture under sonication. MnO_2_-NPs hybrid hydrogel was prepared by exploiting the thermal-triggered sol-gel transition of collagen. The treatment efficacy of hiPSC-EPCs and MnO_2_-NPs hybrid hydrogel was assessed in a hindlimb ischemia mouse model. The protective effect of MnO_2_-NPs on hiPSC-EPCs under oxidative stress was explored via immunofluorescence staining, transcriptome sequencing, Western blotting, enzyme-linked immunosorbent assay, mitochondrial function assays, and quantitative polymerase chain reaction.

**Results:**

In this study, we developed an injectable collagen hydrogel with high clinical translational potential, incorporated with MnO_2_-NPs for the delivery of hiPSC-EPCs. Upon injection, the hydrogel undergoes thermal-triggered gelation, ensuring efficient cell retention at the ischemic site. More importantly, MnO_2_-NPs provide a dual protective function by scavenging extracellular ROS and mitigating intracellular ROS via upregulation of MnSOD in transplanted hiPSC-EPCs. This comprehensive modulation of ROS significantly improves cell survival and functionality. Consequently, the protected hiPSC-EPCs robustly promote angiogenesis, restoring blood perfusion and improving limb salvage in critical limb ischemia.

**Conclusions:**

This MnO_2_-based strategy represents a novel dual-action approach for enhancing cell therapy in ischemic diseases.

## Introduction

Peripheral arterial disease affects over 230 million people worldwide, particularly the elderly, and its end-stage, critical limb ischemia (CLI), is linked to high risks of cardiovascular events, amputation, and mortality 1-5. Currently, endoluminal stenting and artificial vascular bypass are the only feasible surgical treatment method, but they have low long-term patency rates and involve high levels of trauma. However, current therapies fail to address the underlying ischemic pathology of CLI, highlighting the urgent need for novel regenerative strategies.

Stem cell therapy has been recently recognized as one of the most promising approaches. Endothelial progenitor cells (EPCs) are precursor cells found in the bone marrow and peripheral blood that can differentiate into endothelial cells (ECs). EPCs play a crucial role in the endothelial repair of vascular damage by directly differentiating into ECs for re-endothelialization, as well producing vasoactive mediators that exert paracrine and autocrine effects on angiogenesis 6. Some studies have demonstrated that cell therapy utilizing EPCs is both safe and effective for the treatment of CLI 7, 8. However, the clinical application of EPCs is hindered by their scarcity and compromised functionality in patients with comorbidities. A study reveals that the proportion of EPCs in bone marrow is less than 0.01%, while their representation in peripheral blood is similarly limited to only 0.01% 9. Furthermore, in patients with ischemic cardiovascular risk factors such as advanced age, hypertension, or type 2 diabetes, EPCs exhibit impairments in function, quantity, and survival 10-12. Additionally, alterations in circulating EPCs level have been associated with physical frailty 13. Therefore, the identification of effective methods for obtaining a sufficient quantity of functional EPCs constitutes an urgent challenge that necessitates resolution.

Human induced pluripotent stem cell (hiPSC), capable of differentiating into EPCs, offer a renewable and ethically acceptable source 14-17. Several studies have reported that hiPSC-derived EPCs (hiPSC-EPCs) have shown great potential in CLI therapy, yet their clinical translation remains limited 18, 19. A major challenge is the poor survival and retention of transplanted hiPSC-EPCs in the ischemic microenvironment 20-22. Recent studies have shown that biomaterials can improve the ischemic microenvironment by mitigating oxidative stress, primarily through scavenging reactive oxygen species (ROS), thereby enhancing transplanted cell retention and survival 23-26. However, conventional biomaterials primarily scavenge extracellular ROS and fail to address hypoxia-induced intracellular oxidative stress. Hypoxia drives glycolytic shift in transplanted cells, paradoxically increasing intracellular ROS that compromise viability 27, 28.

To overcome the dual challenges of poor cell retention and the hostile oxidative microenvironment, we developed an injectable, ROS-scavenging hybrid hydrogel and demonstrated its ability to eliminate intracellular ROS, providing a dual-function strategy for CLI therapy. This delivery system incorporates MnO_2_ nanoparticles (MnO_2_-NPs) into a type I collagen matrix, enabling simultaneous scavenging of extracellular and intracellular ROS. Unlike conventional scaffolds, MnO_2_-NPs can penetrate cell membranes and neutralize intracellular ROS generated by hypoxia-driven glycolysis. Furthermore, MnO_2_ catalytically decomposes H_2_O_2_ to generate oxygen, thereby mitigating hypoxia and restoring redox balance. Additionally, the collagen matrix provides structural support, promotes the retention of hiPSC-EPCs, and allows for controlled Mn^2+^ release to ensure biosafety 29-31. On the other hand, both MnO_2_-NPs and type I collagen are biocompatible materials with well-documented clinical relevance, supporting their translational potential in therapeutic applications. This dual-action hydrogel system comprehensively modulates the ischemic microenvironment-removing harmful ROS and alleviating hypoxia, thereby significantly enhancing cell survival, paracrine function, and angiogenesis. Ultimately, this strategy offers a potent therapeutic platform for restoring perfusion and promoting tissue regeneration in CLI.

## Materials and Methods

### Synthesis and characterizations of MnO_2_-NPs

MnO_2_-NPs were synthesized following a procedure outlined in previous reports 29. Initially, 1.2 g of polyvinyl pyrrolidone (PVP) was dissolved in 8 mL of distilled water, and then 3 mL of a 20 mg/mL aqueous solution of MnCl_2_ was added to the PVP solution. The resulting mixture was processed to ultrasound treatment for 20 min to ensure complete homogenization. Subsequently, 1 M NaOH solution was dripped to adjust the pH to 12. The mixture was left at ambient temperature for 2 h to facilitate the formation of MnO_2_ nanoparticles. Finally, the product was washed three times with distilled water to remove any impurities and obtain the purified MnO_2_ nanoparticles.

### Fabrication and analysis of MnO_2_-NPs-Gel

The MnO_2_-NPs hybrid hydrogel (MnO_2_-NPs-Gel) was fabricated via thermally induced sol-gel transition of collagen. A solution of type I collagen (Corning, USA) was prepared by dissolving the lyophilized powder in chilled 2% acetic acid, resulting in a homogeneous solution (2 mg/mL). The pH of the solution was adjusted to neutrality, followed by the addition of MnO_2_ nanoparticles (1, 5, 10, and 20 μg/mL), yielding a collagen-MnO_2_-NPs mixture. Following this step, an aliquot of 2 mL from the mixed solution was transferred to a sterile glass vial, followed by incubation at 37 ºC for 1 h. The final concentration of collagen in the prepared hydrogels used for the main experiments was 2 mg/mL. The viscoelastic properties of hybrid hydrogel were examined by loading the collagen-MnO_2_-NPs mixture onto an ARES-RFS rheometer and heating it to 37 ºC. The storage modulus (G′) and loss modulus (G′′) of the hydrogels were evaluated after 1 hour. The microscopic morphology of the hydrogels was detected using scanning electron microscope.

### Evaluation of ROS-scavenging capacity

MnO_2_-NPs were first suspended in aqueous solutions at concentrations of 1, 5, 10, and 20 μg/mL, followed by the addition of H_2_O_2_ (100 μM). After a 10 minutes incubation at ambient temperature, 100 μL of 3, 3', 5, 5'-Tetramethylbenzidine (TMB) solution (10 mg/mL) was incorporated into the mixture. The ROS-scavenging potential of MnO_2_-NPs was determined by observing the color changes of the solution resulting from TMB oxidation. In addition, a peroxide assay kit (Beyotime, China) was used to measure the decomposition of H₂O₂ during incubation with MnO_2_-NPs.

### Determination of critical concentration of collagen hydrogel

The 2 mg/mL collagen hydrogel solution was diluted to a series of concentration gradients (0.125, 0.25, 0.5, 1, 2 mg/mL) in equal proportion, then incubated at 37 ºC. Take it out respectively for 10, 30, and 60 minutes to observe whether hydrogel gelation was complete.

### Determination of gelation time of collagen hydrogel

The collagen hydrogels at concentrations of 0.125, 0.25, 0.5, 1, and 2 mg/mL were incubated at 37 ºC, and was taken out at 0, 2, 5, 10 and 60 minutes to observe whether hydrogel gelation was complete.

### Cell culture

We employed three hiPSC lines. These cells were maintained on plates coated with Matrigel (Corning, USA) in TeSR-E8 medium (STEMCELL, Canada), and the cells were passaged by using ReLeSR (STEMCELL, Canada).

### EPC differentiation

The protocol for EPC differentiation is based on previous study and has been appropriately adjusted 19. Once the cells reach 80-90% confluence, dissociate them into single-cells suspensions using Accutase (Thermo Fisher, USA). Next, seed the cells at a density of 50,000 cells/cm^2^, then maintain them in E8 medium containing 10 ng/mL LY83583 (MedChemExpress, USA) for 24 h. On day 0, the predifferentiation medium was replaced with the DMEM/F12 (Gibco, USA) supplemented with 60 μg/mL ascorbic acid (Sigma Aldrich, USA), 3 μM CP21R7 (MedChemExpress, USA), 25 ng/mL BMP4 (NovoProtein, China) and 50 ng/mL FGF2 (NovoProtein, China) at a volume of 2 mL/10 cm^2^. On day 1, the medium was exchanged for DMEM/F12 medium with 60 μg/mL ascorbic acid, 25 ng/mL BMP4 and 50 ng/mL FGF2 at a volume of 2 mL/10 cm^2^. On day 2, the prior medium was substituted with DMEM/F12 medium enriched with 200 ng/mL VEGF165 (NovoProtein, China), 10 μM DAPT (GLPBIO, USA), 2 μM forskolin (GLPBIO, USA), using 2 mL/10 cm^2^ of culture. On days 3 and 4, the medium was refreshed. On day 5, the differentiated cells were harvested, and a portion was analyzed by FACS for endothelial markers, including CD34, CD144, CD31, and VEGFR2 (Table S1). Subsequently, the purified cells were and maintain in 2 mL ECM medium (ScienCell, USA). The human umbilical vein endothelial cells (HUVECs) were cultured using the same method as hiPSC-EPCs.

### Cell proliferation

5000 HUVECs were plated in per well of a 96-well plate and cultured with 100 μL of ECM medium. In addition, hiPSC-EPCs were seeded in a 0.4 μm transwell chambers (5000 cells/ well) and co-cultured with HUVECs. Subsequently, the medium was replaced, and 10 μL of Cell Counting Kit-8 (CCK8) solution (GLPBIO, USA) was added to each well following 24 h, 48 h, or 72 h co-culture. Upon incubation at 37 ºC for 1 h, the results were quantified using a multi-mode microplate reader (Tecan Infinite F200 Pro, Switzerland).

### Wound healing

HUVECs were cultured in 6-well plate. Once the cells reached 90% confluence, a straight wound was created using a pipette tip and recorded under a microscope. Subsequently, hiPSC-EPCs were seeded in 0.4 μm transwell chambers (5 X 10^6^ cells/ well). Upon completion of the 24 h co-culture period, the transwell chambers were removed and wound healing was detected using a microscope (Leica DMi8, Germany).

### Tube formation

HUVECs were plated in 12-well plate (1 X 10^6^ cells/ well) that has been treated with matrigel for 30 min. Subsequently, hiPSC-EPCs were seeded in a 0.4 μm transwell chamber (1 X 10^6^ cells/ well). After co-culturing for 6 h, the transwell chamber was removed and tube formation was detected under a microscope. The results of newly formed sprouting tubes were analyzed using software ImageJ.

### Quantitative polymerase chain reaction (q-PCR)

The RNA extraction from hiPSC, hiPSC-EPCs, or HUVECs was conducted with RNAzol reagent (Molecular Research Center, USA), followed by reverse transcription of 1 μg RNA into cDNA using the Superscript First-Strand cDNA Synthesis Kit (NovoProtein, China). The q-PCR result was detected by a LightCycler 480 II System (Roche, Switzerland) with the SYBR Premix Ex Taq II kit (Vazyme, China). The primer sequences for q-PCR are provided in Table S2.

### Western blot

Proteins were isolated from cells by using a lysis buffer based on radioimmunoprecipitation assay (Thermo Fisher, USA), which was supplemented with a cocktail of protease inhibitors (Phygene, China) and 1 mM PMSF (Thermo Fisher, USA). The proteins of equal quality were then loaded onto an SDS-PAGE gel (BioRad, USA) for electrophoretic separation. After the proteins were transferred to a PVDF membrane, the blots were blocked with 5% BSA dissolved in TBST. Subsequently, the membranes were incubated with primary antibodies against CuZnSOD, MnSOD, β-actin, p-NF-κB, NF-κB, p-IκBα, IκBα and GAPDH, followed by treatment with a secondary HRP-conjugated antibody (anti-rabbit/mouse IgG HRP-linked antibody) (Table S1). Protein expression levels were assessed using a chemiluminescence detection system (NCM Biotech, China).

### Characterization of cell types

HiPSCs or hiPSC-EPCs were maintained in 6-well plates with 2 mL of ECM medium. Following incubation, the cells were trypsinized and individually labeled with antibodies against SOX2, OCT4, Nanog, CD34, CD144, CD31, or VEGFR2 for 30 min (Table S1). After a triple rinse in PBS, the staining of these markers was analyzed using a CytoFLEX system (Beckman Coulter, USA).

### Cytotoxicity

5000 HUVECs or hiPSC-EPCs were plated per well in a 96-well plate and maintained in 100 μL of ECM medium for 24 h. Then, the medium was replaced with 100 μL of fresh ECM. MnO_2_-NPs dispersed in PBS were added to the wells, and the cells were treated at 37 ℃ for 72 h. Following incubation, the medium was discarded, and 10 μL of CCK8 solution (GLPBIO, USA) was added. After a 1-h incubation at 37 ºC, absorbance was quantified using a multi-mode microplate reader.

### Live-dead assay

MnO_2_-NPs-Gel was synthesized using the previously reported method and subsequently placed into 6-well plates. After stable hydrogel formation, 50,000 hiPSC-EPCs were seeded onto the hydrogel. Following a 24-h incubation, the medium was replaced with fresh medium containing either 4 μM LY83583 or 100 μM H_2_O_2_. After incubation for 48 h, calcein AM (MedChemExpress, USA) and propidium iodide (PI) (Tobon, USA) were added. The staining cells were examined using an LSM 880 fluorescence microscope (Zeiss, Germany).

### ROS-induced apoptosis analysis

HUVECs or hiPSC-EPCs were seeded at a density of 50,000 cells/mL onto MnO₂-NPs-Gel contained within a round dish. Following a 24-h incubation, the original medium was substituted with a new medium that contained either 4 μM LY83583 or 100 μM H_2_O_2_. Following a 48-h incubation, the cells were trypsinized and stained with Annexin V and 7-AAD for flow cytometry analysis (Tonbo Biosciences, USA).

### Detection of Superoxide Dismutase Activity

The enzymatic activity of superoxide dismutase in hiPSC-EPCs and HUVECs was detected using a CuZnSOD/MnSOD activity assay kit (Elabscience, China). One million cells were collected and homogenized, after which the supernatant was carefully collected to determine protein concentration. The samples were assayed for enzymatic activity using a commercially available kit following the manufacturer's protocol.

### Measurement of mitochondrial membrane potential (MMP)

A commercially available JC-1 kit (Beyotime, China) was employed to assess MMP across various treatment conditions by measuring the red-to-green fluorescence ratio. Briefly, after 48 h of treatment with 4 μM LY83583 or 10 μg/mL MnO_2_-NPs, hiPSC-EPCs were processed following the manufacturer's protocol. Then, the cells were visualized with a fluorescence microscope.

### Transcriptome sequencing

HiPSC-EPCs were incubated under different conditions for 48 h, including the Control group, the LY83583 treatment group, and the LY83583 + MnO₂-NPs treatment group. Then, cells were collected, and the total RNA was extracted using RNAzol reagent. Finally, the above cell samples were sent to Yiyue Biotech (Guangzhou, China) for transcriptome sequencing analysis.

### Animals

The animal experiments were conducted in accordance with the guidelines approved by the Animal Care and Use Committee at Sun Yat-sen University (SYSU-IACUC- 2024-001482). Every effort was made to alleviate discomfort, with the application of the 3Rs (Replacement, Reduction, and Refinement) throughout the study. BALB/c nude mice, aged 6-8 weeks, were sourced from Beijing Vital River Laboratory Animal Technology Co., Ltd. The mice were allowed a minimum of one week for acclimatization before the experiments began. Following surgical procedures, the mice were placed in recovery boxes maintained at 37 °C and were closely monitored for a minimum of 7 days during the recovery phase.

### Murine hindlimb ischemia model

BALB/c nude mice received inhalation anesthesia using 1% isoflurane. To induce limb ischemia injury, a midline abdominal incision was made, and the distal common iliac artery along with the origin of the epigastric artery was ligated using sterile methods. After the procedure, the mice were randomly allocated to the designed experimental groups.

### Analysis of post-transplantation cell engraftment

Mice with limb ischemia were randomly assigned to 4 groups (three mice per group), after which they received intramuscular injections of one of the following treatments: HUVECs, hiPSC-EPCs, hiPSC-EPCs delivered by Collagen-Gel or hiPSC-EPCs delivered by MnO_2_-NPs-Gel. Each mouse was administered 100 μL of hiPSC-EPCs suspension (1 × 10^6^ cells/mL) or an equivalent volume of hydrogel containing the cells, with injections delivered intramuscularly at three sites within the adductor muscle. The cells were pre-labeled with CM-Dil before the injection. At one-week post-injection, the mice were euthanized, and the impaired limbs were harvested. Subsequently, the tissues were fixed in 4% paraformaldehyde, embedded in paraffin, sectioned, and histologically examined. To assess the viability of the implanted cells, the percentage of positively stained cells (red fluorescence) was quantified across five randomly chosen microscopic fields.

### Evaluation of limb function and ischemic injury

The ischemia model mice were randomly assigned into 7 groups (*n* = 11) and received intramuscular injections of either PBS, Collagen-Gel, MnO_2_-NPs-Gel, HUVECs, hiPSC-EPCs, hiPSC-EPCs delivered by Collagen-Gel or hiPSC-EPCs delivered by MnO_2_-NPs-Gel. Each mouse was received 100 μL of therapeutic cells (1 × 10^7^ cells/ mL) or an equal volume of hydrogel with the cells, injected into three sites in the adductor muscle. Following injection, hindlimb blood perfusion was assessed using a laser Doppler imager (Perimed, Sweden) on days 1, 3, 7, 14, 21, and 28. Ischemic injury was assessed according to a previously described method (0, no skin tone change; 1, slight skin tone change; 2, moderate to severe skin tone change; 3, foot necrosis; 4, dragging of foot). Limb function was assessed using a scoring system based on previous reports (0, toes can bend; 1, the sole of the foot can bend; 2, not limping; 3, limping).

### Histology

Following treatment with PBS, Collagen-Gel, MnO_2_-NPs-Gel, HUVECs, hiPSC-EPCs, hiPSC-EPCs delivered by Collagen-Gel or hiPSC-EPCs delivered by MnO_2_-NPs-Gel, the mice were euthanized and the ischemic limbs were harvested. Subsequently, the tissues were treated with 4% paraformaldehyde for fixation, encapsulated in paraffin, sliced into sectioned, and then subjected to staining with hematoxylin-eosin (H&E) and Masson's trichrome. Finally, the sections were examined using a microscope.

### Immunofluorescence (IF) analysis

IF analysis was conducted on OCT-embedded frozen tissue slices to assess the *in vivo* angiogenic potential of implanted cells. The tissue sections were incubated with 0.2% Triton X-100, subsequently blocked with 10% goat serum, and then incubated overnight at 4 °C with primary antibodies. Afterward, the samples were incubated with secondary antibodies conjugated to Alexa Fluor 488 or 594 for 40 min at room temperature in the dark. Nuclei were labeled with 4',6-diamidino-2-phenylindole (DAPI). The sections were visualized using a confocal microscope. The expression of CD31 was quantified to determine the proangiogenic activity of the implanted stem cells (Table S1).

### Statistical analysis

All results are based on data obtained from a minimum of three separate experiments. Statistical analyses were conducted using Student's *t* test, one-way analysis of variance (ANOVA), or multivariate ANOVA as appropriate. *p < 0.05 was regarded as indicative of statistical significance. The data are presented as the means ± *SD*.

## Results

### Preparation and characterization of hiPSC-EPCs

**Figure 1:**
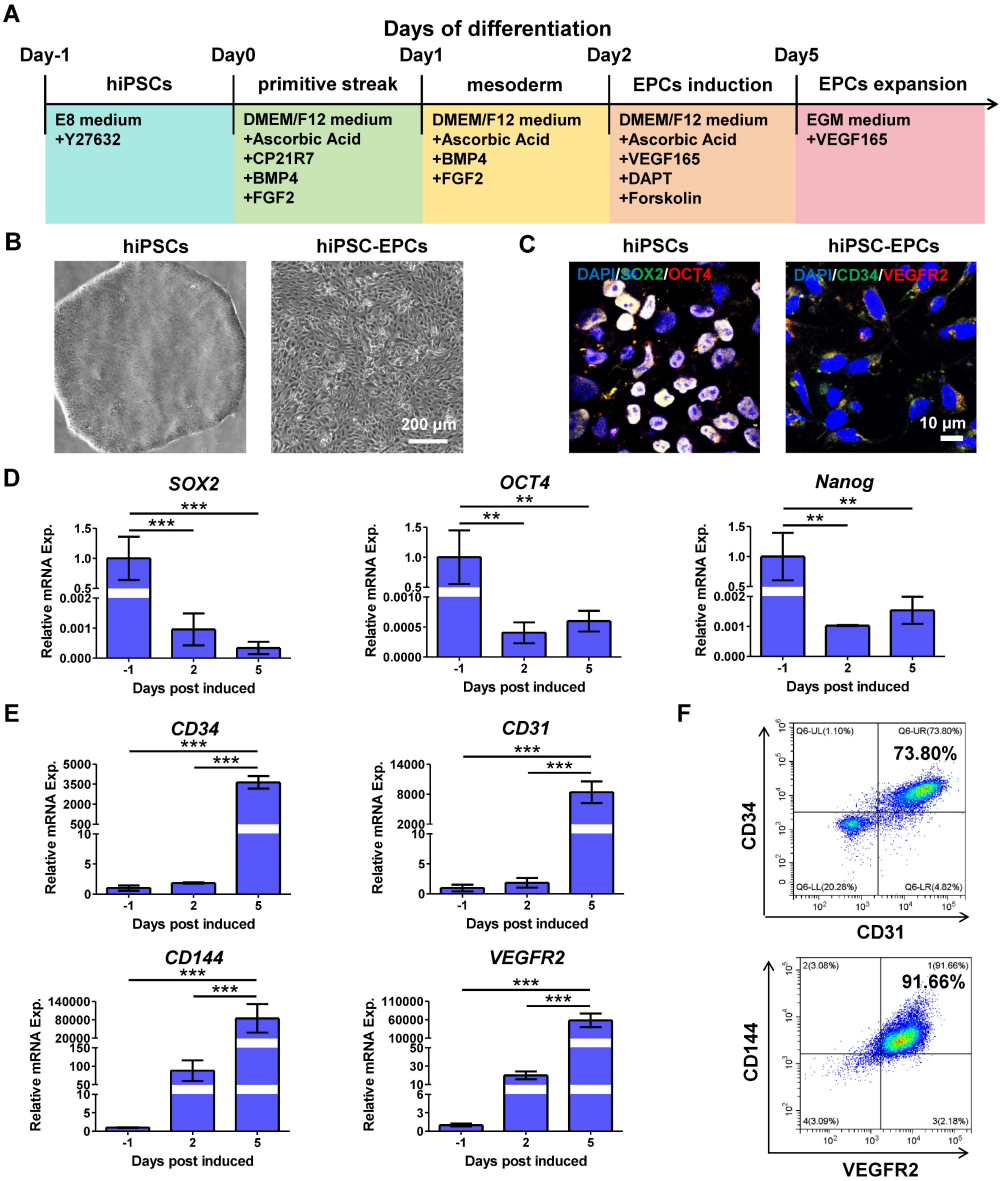
**Characterization of hiPSCs or hiPSC-EPCs.** (**A**) Schematic representation of the differentiation conditions used to generate hiPSC-EPCs from hiPSCs. (**B**) Morphological characterization of hiPSCs and hiPSC-EPCs. Scale bar: 200 μm. (**C**) HiPSCs and hiPSC-EPCs were verified by staining with typical IPS markers SOX2, OCT4 or EPC surface markers CD34 and VEGFR2. Scale bar: 10 μm. (**D**) The relative mRNA expressions of *SOX2*, *OCT4* and *Nanog* in the induced cells were detected by q-PCR (*n* = 3). (**E**) The relative mRNA expressions of *CD34*, *CD144*, *CD31* and *VEGFR2* in the induced cells were detected by q-PCR (*n* = 3). (**F**) Co-expression of CD34/CD31 or CD144/VEGFR2 for hiPSC-EPCs was detected by FACS. The data represent mean ± *SD*. *ns* = no significance, *p < 0.05, **p < 0.01, ***p < 0.001, by one-way ANOVA.

To investigate a non-invasive source of EPCs, we updated a method of EPC differentiation, which effectively generates hiPSC-EPCs from hiPSCs with high efficacy. Compared with previous EPC differentiation schemes in our laboratory, the scheme proposed here refers to other studies to add several growth factors and small molecules to enhance the differentiation efficiency 19, 32. This protocol consists of three distinct phases characterized by the specific medium composition that transitions hiPSCs first to the primitive streak, subsequently to mesoderm, and ultimately to primitive endothelium (Figure 1A). Using this differentiation protocol, hiPSCs (Figure 1B, left) were effectively transformed into hiPSC-EPCs (Figure 1B, right). The induced cells expressed hiPSC markers (SOX2, OCT4) on Day 0 of differentiation and EPC surface markers (CD34, VEGFR2) on Day 5 (Figure 1C). Q-PCR results indicated that the expression levels of hiPSC markers *SOX2*, *OCT4* and *Nanog* decreased following differentiation (Figure 1D). On the contrary, the expression of EPC markers (*CD34*, *CD31*, *CD144* and *VEGFR2*) increased during the process (Figure 1E). Compared to the original differentiation protocol, flow cytometry analysis confirmed the more efficient and successful differentiation of EPCs, as indicated by CD34/CD31 and CD144/VEGFR2 double positive rates (Figure 1F, Figure S1A-B). In addition, pluripotent stem cells derived from other cell lines were also efficiently induced into EPCs using the improved differentiation protocol (Figure S1C-D).

Some studies have demonstrated that EPCs can promote the repair of injured blood vessels by mediating the proliferation and migration of ECs 33, 34. To verify the ability of hiPSC-EPCs to mediate vascular repair, the CCK8 assay was employed to assess the proliferation of HUVECs co-cultured with hiPSC-EPCs. The viability and proliferation of HUVECs were enhanced after co-culture with hiPSC-EPCs compared to the control (Figure S2A). Then, we investigated the effect of hiPSC-EPCs on the migration of HUVECs by employing the wound healing method. HiPSC-EPCs enhanced the migratory ability of HUVECs relative to the control (Figure S2B-C). After treating HUVECs with hiPSC-EPCs, *in vitro* angiogenesis was evaluated through tube formation. It can be clearly evident that hiPSC-EPCs considerably strengthened the angiogenic potential of HUVECs. A notably higher quantity of newly formed sprouting tubes was detected after co-culture with hiPSC-EPCs compared to the HUVECs-only group (Figure S2D-E). These results show that the obtained hiPSC-EPCs exhibit the characteristic of promoting angiogenesis.

### Synthesis and characterization of MnO_2_-NPs-Gel

**Figure 2:**
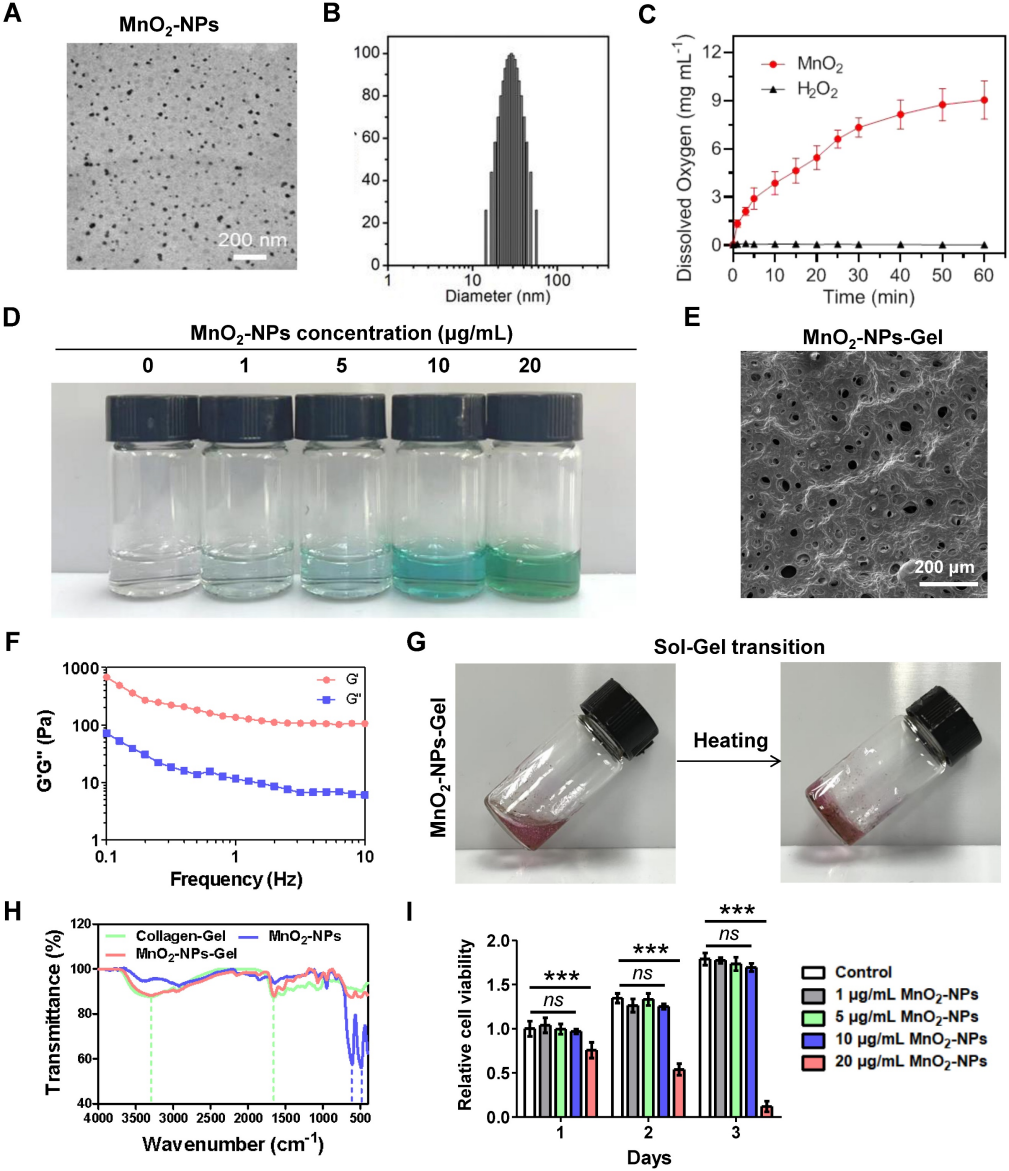
**Characterization of MnO_2_-NPs-Gel.** (**A**) Transmission electron microscopy (TEM) image of MnO_2_-NPs in aqueous solution. (**B**) Size distribution of MnO_2_-NPs in aqueous solution. (**C**) Dissolved oxygen produced by co-incubation of H_2_O_2_ with MnO_2_-NPs. (**D**) Optical image of 100 μM H_2_O_2_ aqueous solution incubated with different concentrations of MnO_2_-NPs (0, 1, 5, 10 and 20 μg/mL) for 10 min followed by addition of HRP and TMB. (**E**) Interior morphology of the representative MnO_2_-NPs-Gel was observed by scanning electron microscopy. Scale bar: 200 μm. (**F**) Rheological properties of MnO_2_-NPs-Gel were analyzed with frequency changes by rheological measurements. (**G**) Thermal-triggered sol-gel transition of the mixture of MnO_2_-NPs and Collagen-Gel. (**H**) Characteristic transmittance peak of MnO_2_-NPs, Collagen-Gel and MnO_2_-NPs-Gel were detected by fourier-transform infrared. (**I**) Cytotoxicity of MnO_2_-NPs at different concentrations was analyzed using a CCK-8 kit. The quantified data represent the cell viability normalized to the control group of day 1 (*n* = 3). The data represent mean ± *SD*. *ns* = no significance, *p < 0.05, **p < 0.01, ***p < 0.001, by two-way ANOVA.

To prepare highly water-dispersible MnO_2_-NPs, MnO_2_ was dissolved in NaOH aqueous solution and neutralized under sonication. The synthesized MnO_2_-NPs have a spherical shape with an average size of approximately 30 nm and exhibit strong stability (Figure 2A-B). The visual inspection of MnO_2_-NPs revealed a brown color (Figure S3A). After successfully synthesizing MnO_2_-NPs, we assessed their ability to scavenge ROS by incubating them with 100 μM of H_2_O_2_, a concentration similar to ROS levels in critical ischemic tissues 35. The measured dissolved oxygen gradually increased over time, confirming that MnO_2_-NPs can catalyze H_2_O_2_ to generate oxygen (Figure 2C). Further investigation demonstrated that MnO_2_-NPs have a dose-dependent effect in catalyzing H_2_O_2_ to produce oxygen, which subsequently oxidizes colorless TMB to a deep blue color (Figure 2D) 36. In addition, experimental results from the peroxide assay kit demonstrated that MnO_2_-NPs can effectively catalyze H_2_O_2_ decomposition in a dose-dependent manner (Figure S3B).

Once the ROS-scavenging properties of MnO_2_-NPs were validated, we incorporated these nanoparticles into an injectable hydrogel. The injectable hydrogel exhibited a burgundy color, and was constructed using type I collagen, which is broadly applied as scaffolds in cell delivery (Figure S3C) 37-40. The hydrogels exhibit the characteristic microscopic morphology commonly seen in traditional collagen-based hydrogels that may serve as an extracellular matrix, imitating the niche for the attachment of MnO_2_-NPs and therapeutic cells (Figure 2E) 39. In viscoelastic analysis, the storage modulus (G') is found to be greater than the loss modulus (G''), which indicates the characteristic solid-like rheology of hydrogels (Figure 2F) 41. Subsequently, the hydrogel undergoes a thermal-triggered sol-gel transformation at 37 ℃ (Figure 2G) 42. Notably, the 2 mg/mL collagen hydrogel solution formed a stable gelation within 5 min at 37 ºC, while the 1 mg/mL collagen solution at the critical gelation concentration required approximately 60 minutes to complete the gelation under identical conditions (Figure S3D-E). Furthermore, we suspended hiPSC-EPCs in collagen hydrogel and seeded the mixture into the upper chamber of a transwell insert; the lower chamber was pre-coated with matrigel and seeded with HUVECs to test hiPSC-EPC paracrine pro-angiogenic capacity in 3D. Both the hiPSC-EPCs alone and hiPSC-EPCs/Collagen-Gel groups enhanced HUVEC tube formation versus hydrogel-only controls, with no difference between them. These findings indicate that collagen hydrogel does not impair hiPSC-EPC paracrine function (Figure S3F-G). Fourier-transform infrared (FTIR) spectroscopy confirmed the successful incorporation of MnO_2_-NPs into hydrogel with the characteristic transmittance peak of MnO_2_-NPs was observed at 486 cm^-1^ and 609 cm^-1^, and that of the hydrogel at 1656 cm^-1^ and 3296 cm^-1^(Figure 2H) 43,44. Inductively coupled plasma mass spectrometry (ICP-MS) analysis demonstrated that MnO₂-NPs were internalized by hiPSC-EPCs (Figure S3H). Cell compatibility reflects the toxic effects of MnO_2_-NPs at concentrations of 1, 5, 10 and 20 μg/mL, while 20 μg/mL MnO_2_-NPs caused cell apoptosis, indicating that the appropriate concentration of MnO_2_-NPs is non-cytotoxic (Figure 2I) 45-46.

### MnO_2_-NPs enhanced cell survival in oxidative stress injury induced by reactive oxygen species

**Figure 3:**
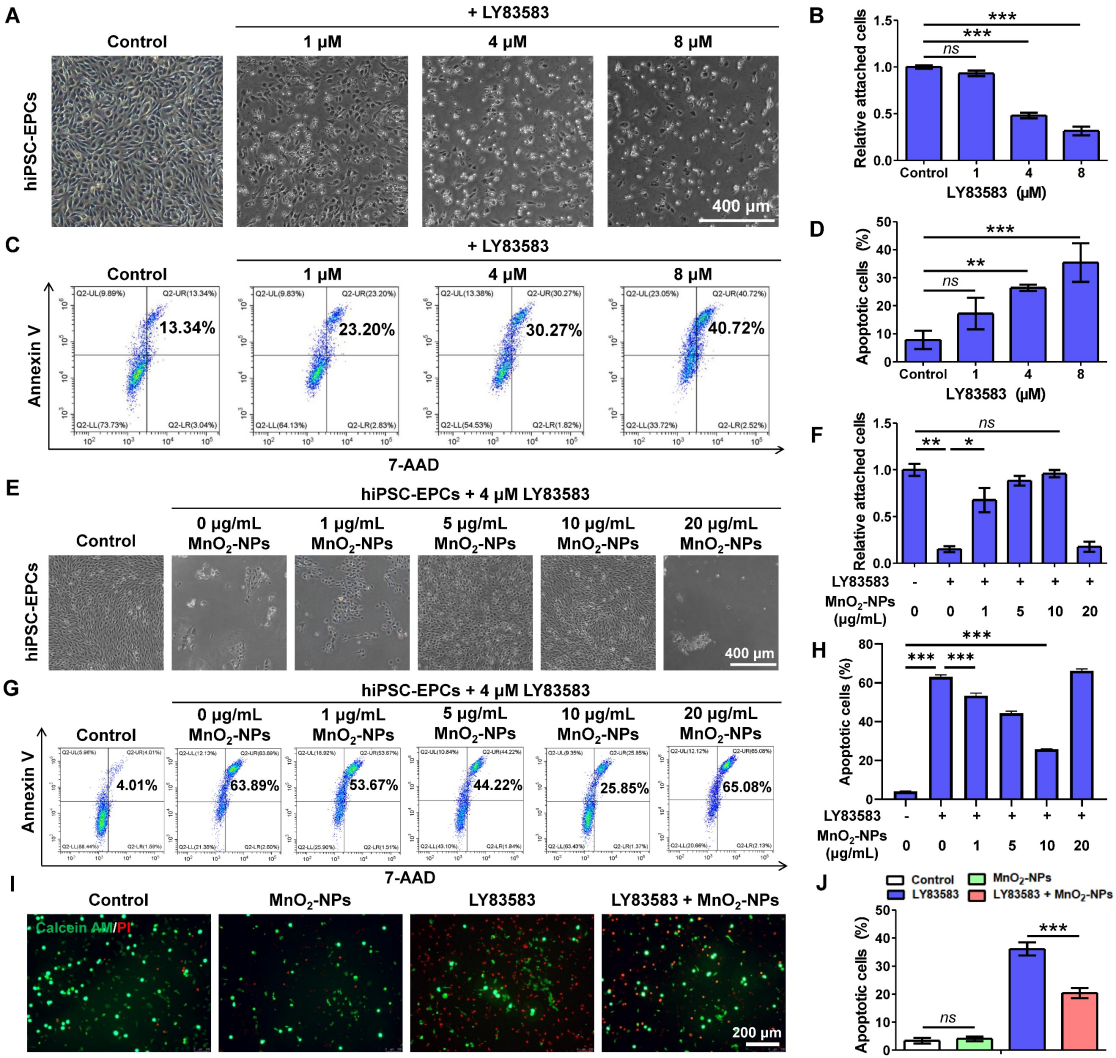
** The capacity of MnO_2_-NPs to enhance cell survival *in vitro*.** (**A-B**) Morphological characteristics (**A**) and quantitative analysis (**B**) of attached hiPSC-EPCs incubated with 1, 4 or 8 μM LY83583 for 48 h (*n* = 3). Scale bar: 400 μm. (**C-D**) Apoptotic cells (**C**) and quantitative analysis (**D**) of hiPSC-EPCs incubated with 1, 4 or 8 μM LY83583 for 48 h were detected by annexin-V and 7-AAD staining using FACS (*n* = 3). (**E-F**) Morphological characteristics (**E**) and quantitative analysis (F) of hiPSC-EPCs treated with 4 μM LY83583 and 1, 5, 10 or 20 μg/mL MnO_2_-NPs for 48 h. Scale bar: 400 μm (*n* = 3). (**G-H**) Apoptotic cells (**G**) and quantitative analysis (H) of hiPSC-EPCs treated with 4 μM LY83583 and 1, 5, 10 or 20 μg/mL MnO_2_-NPs for 48 h (*n* = 3). (**I-J**) Representative images (**I**) and quantitative analysis (J) of live-dead assay of hiPSC-EPCs incubated with 4 μM LY83583 and 10 μg/mL MnO_2_-NPs for 48 h. The live cells were stained with calcein AM as green fluorescence and dead cells were stained with propidine iodide (PI) as red fluo rescence (*n* = 3). Scale bar: 200 μm. The data represent mean ± *SD*. *ns* = no significance, *p < 0.05, **p < 0.01, ***p < 0.001, by one-way ANOVA.

After successfully synthesizing MnO_2_-NPs and confirming their ROS-scavenging capability, we subsequently assessed their potential to act as biomimetic scaffolds for improving the survival of therapeutic cells. LY83583, a generator of superoxide anions (O_2_^-^) in living cells, was used to induce oxidative stress damage to hiPSC-EPCs 47, 48. Severe cytotoxicity was observed in hiPSC-EPCs treated with 1, 4 or 8 μM LY83583, characterized by cell deformation, detachment, formation of cell debris and apoptosis (Figure 3A-D). Further investigation revealed that hiPSC-EPCs exhibited greater resistance to injury induced by LY83583 compared to HUVECs (Figure S4A-D). Based on the dose-dependent cytotoxicity of LY83583, a concentration of 4 μM was selected for subsequent experiments. Subsequently, hiPSC-EPCs were incubated with 4 μM LY83583 and 1, 5, 10 or 20 μg/mL MnO_2_-NPs. We observed that MnO_2_-NPs at concentrations of 1, 5 and 10 μg/mL exhibited dose-dependent protective effects on hiPSC-EPCs against cytotoxic reactions induced by LY83583 (Figure 3E-F). Similar result was observed in the flow cytometry analysis. The apoptotic percent of hiPSC-EPCs cultured without MnO_2_-NPs reaches 54.9 ± 13.0%. In comparison, due to the MnO_2_-NPs that abolish ROS, more than 75.2 ± 4.4% of hiPSC-EPCs cultured with 10 μg/mL MnO_2_-NPs remained alive following the same duration of LY83583 treatment (Figure 3G-H). Moreover, the MnO_2_-NPs-Gel demonstrated the comparable cytoprotective efficacy to MnO_2_-NPs, significantly attenuating LY83583-induced apoptosis in hiPSC-EPCs (Figure S5A-B). The collagen hydrogel did not affect the ROS scavenging function of MnO_2_-NPs. Similarly, calcein AM/PI staining results further confirmed that a significant number of apoptotic hiPSC-EPCs stained with red fluorescence after 48 h of treatment with LY83583, and the morphology of most residual cells collapsed. In contrast, the MnO_2_-NPs for ROS scavenging enhances the ability of hybrid hydrogel to promote the survival of hiPSC-EPCs incubated with LY83583 (Figure 3I-J). Besides LY83583, the MnO_2_-NPs also show strong ability to enhance the cell survival in oxidative stress damage induced by H_2_O_2_ (Figure S5C-F).

### MnO_2_-NPs protected mitochondrion from oxidative stress damage

**Figure 4:**
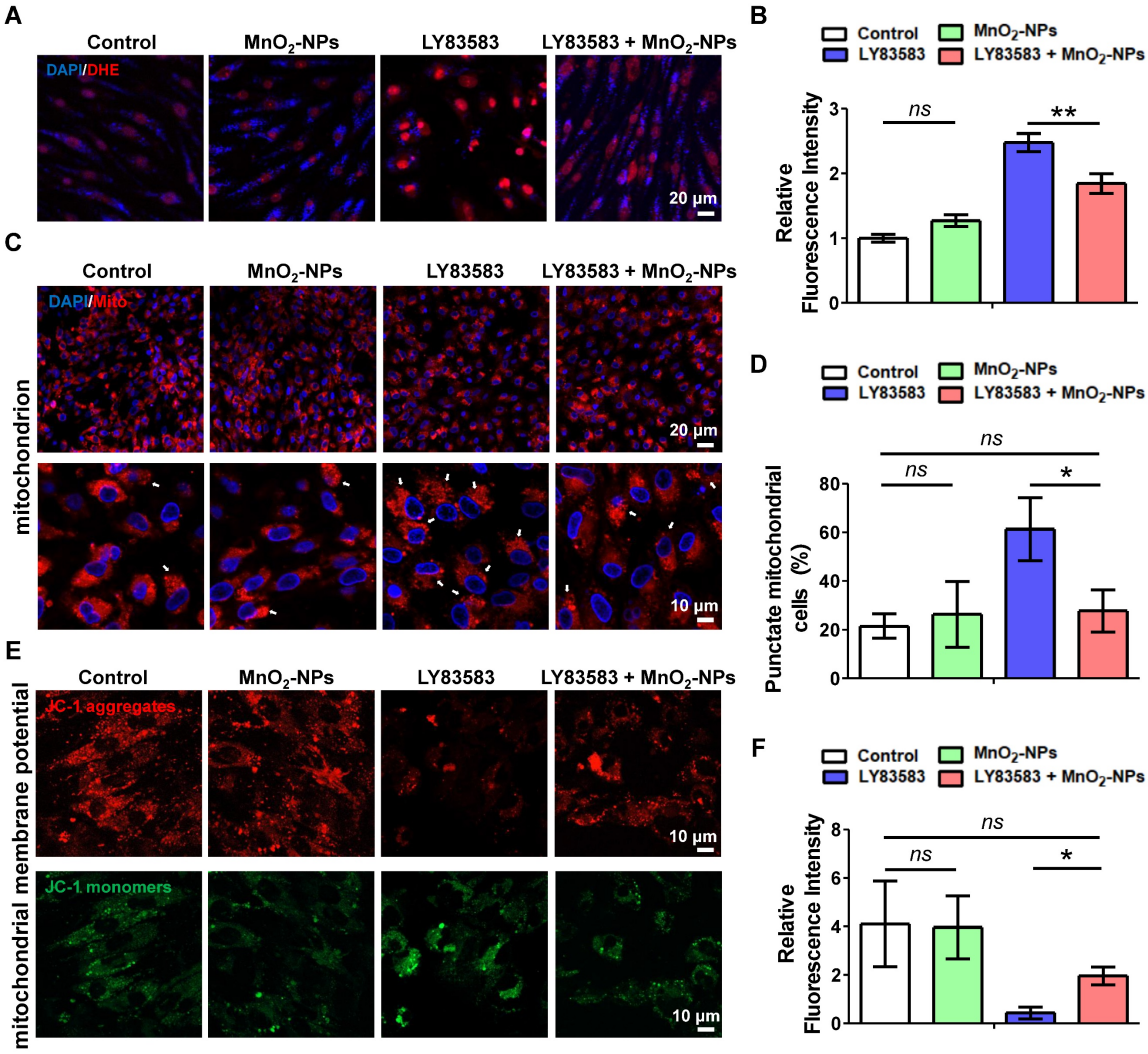
** The capacity of MnO_2_-NPs to protect mitochondrion from oxidative stress damage.** (**A-B**) Representative images (**A**) and quantitative analysis (**B**) of ROS in hiPSC-EPCs incubated with 4 μM LY83583 and 10 μg/mL MnO_2_-NPs for 48 h (*n* = 3). Scale bar: 20 μm. (**C-D**) Representative images (**C**) and quantitative analysis (**D**) of mitochondria in hiPSC-EPCs incubated with 4 μM LY83583 and 10 μg/mL MnO_2_-NPs for 48 h (*n* = 3). The white arrows indicate the cells with punctate mitochondrial appearance (punctate cells). Top scale bar: 20 μm, bottom scale bar: 10 μm. (**E-F**) Representative images (**E**) and quantitative analysis (**F**) of mitochondrial membrane potential of hiPSC-EPCs incubated with 4 μM LY83583 and 10 μg/mL MnO_2_-NPs for 48 h (*n* = 3). Scale bar: 10 μm. The data represent mean ± *SD*. *ns* = no significance, *p < 0.05, **p < 0.01, ***p < 0.001, by one-way ANOVA.

Intracellular ROS levels of hiPSC-EPCs treated with LY83583, both in the presence and absence of MnO_2_-NPs, were detected by the superoxide anion fluorescent probe dihydroethidium (DHE) 49. Compared with hiPSC-EPCs cultured alone, a significant increase in red fluorescence labeled superoxide anion was observed in hiPSC-EPCs incubated with LY83583. Moreover, the addition of MnO_2_-NPs inhibited superoxide anion production induced by LY83583 (Figure 4A-B). Cellular ROS generation occurs either via oxidase enzyme activity or through the dismutation of O_2_^-^, while the constitutive presence of O_2_^-^ results from the leakage in the mitochondrial respiratory chain 50. Therefore, mitochondria are the main generator of ROS in living cell. Further mitochondrial staining demonstrated an intracellular distribution of mitochondria in hiPSC-EPCs with a characteristic thread-like network appearance. Exposure to 4 μM LY83583 for 48 h, 61.3 ± 12.9% of hiPSC-EPCs exhibited the disrupted mitochondrial thread-like networks with the significant punctate appearance. In contrast, under the same stress, only 27.7 ± 8.6% of hiPSC-EPCs incubated with MnO_2_-NPs demonstrated mitochondrial alteration and punctuation (Figure 4C-D). JC-1 was observed to aggregate in the matrix of mitochondria of hiPSC-EPCs and form a red fluorescent polymer, indicating that the MMP of hiPSC-EPCs was relative normal. However, in hiPSC-EPCs treated with LY83583 for 48 h, the green fluorescent JC-1 monomer could not aggregate in the mitochondrial matrix, providing evidence of mitochondrial depolarization 51. Similarly, MnO_2_-NPs protected the MMP of hiPSC-EPCs from oxidative stress damage induced by LY83583 (Figure 4E-F).

### MnO_2_-NPs increasesd the expression and activity of MnSOD in hiPSC-EPCs

**Figure 5:**
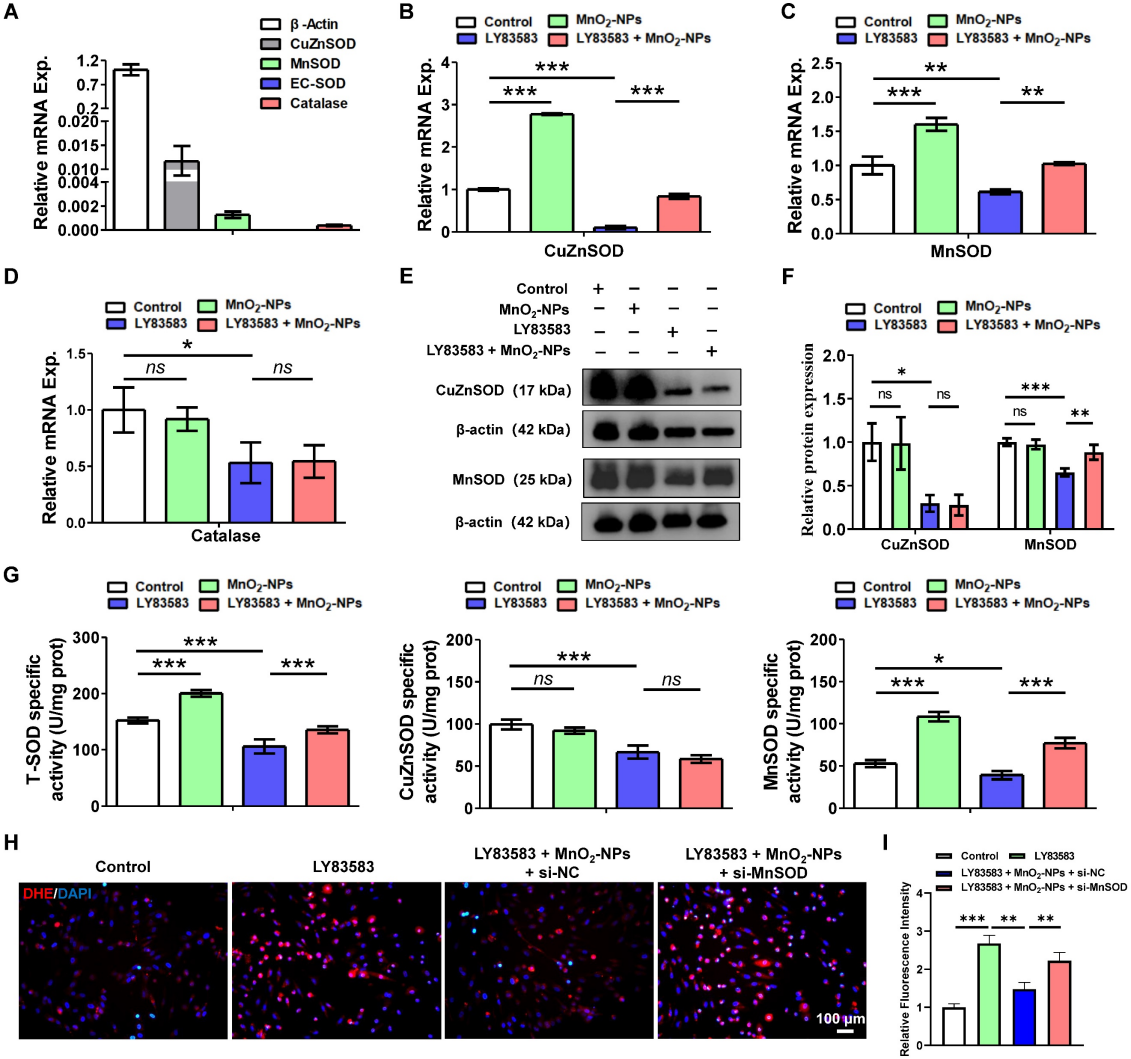
** The capacity of MnO_2_-NPs to enhance the expression and activity of MnSOD.** (**A**) The mRNA levels of CuZnSOD, MnSOD, EC-SOD and catalase in hiPSC-EPCs were detected by q-PCR (*n* = 3). (**B-D**) The mRNA levels of CuZnSOD, MnSOD and catalase in hiPSC-EPCs incubated with 4 μM LY83583 and 10 μg/mL MnO_2_-NPs for 48 h was detected by q-PCR (*n* = 3). (**E-F**) Western blot analysis of CuZnSOD and MnSOD (*n* = 3). (**G**) Enzymatic activity of total SOD, CuZnSOD and MnSOD in hiPSC-EPCs incubated with 4 μM LY83583 and 10 μg/mL MnO_2_-NPs for 48 h (*n* = 3). (**H-I**) Representative images (**H**) and quantitative analysis (**I**) of ROS in hiPSC-EPCs incubated with 4 μM LY83583, 10 μg/mL MnO_2_-NPs and 100 nM siRNA for 48 h (*n* = 3). Scale bar: 100 μm. The data represent mean ± *SD*. *ns* = no significance, *p < 0.05, **p < 0.01, ***p < 0.001, by one-way ANOVA.

ROS are produced as the result of mitochondrial respiration and metabolism, or through the action of specific enzymes such as superoxide dismutases, catalase, and myeloperoxidases 52. Superoxide dismutases (SOD) are a ubiquitous enzymes family that facilitate the dismutation of superoxide anions. SOD1, also known as CuZnSOD is predominantly located in intracellular cytoplasmic spaces. SOD2, or MnSOD is specifically targeted to the mitochondrial spaces. SOD3, referred to as EC-SOD directs exclusively to extracellular regions 53. To elucidate the effects of MnO_2_-NPs on SOD family, we investigated the expression in hiPSC-EPCs. Q-PCR results showed that hiPSC-EPCs expressed *CuZnSOD*, *MnSOD* and *catalase*, but almost did not express *EC-SOD* (Figure 5A). In addition, the mRNA levels and enzyme activities of *CuZnSOD* and *MnSOD* in hiPSC-EPCs were higher than those in HUVEC (Figure S4E, S4F). Further research showed that the mRNA expression of *CuZnSOD*, *MnSOD* and *catalase* in hiPSC-EPCs treated with LY83583 was significantly reduced. On the contrary, MnO_2_-NPs increased *CuZnSOD* and *MnSOD* expression in hiPSC-EPCs and protected them from damage by LY83583, but had no effect on catalase expression (Figure 5B-D). Similarly, LY83583 adversely affected damaged the protein expression levels of CuZnSOD and MnSOD in hiPSC-EPCs, while MnO_2_-NPs still provided protective effects against the damage induced by LY83583 (Figure 5E-F). However, according to the results of enzyme activity, although the enzyme activities of total SOD, CuZnSOD and MnSOD in hiPSC-EPCs were significantly reduced after LY83583 treatment, MnO_2_-NPs did not enhance the enzyme activity of CuZnSOD, but only increased that of MnSOD, indicating that MnO_2_-NPs mainly protects cells from oxidative stress damage by increasing the activity of MnSOD in mitochondria (Figure 5G). Furthermore, MnSOD expression was knocked down in hiPSC-EPCs via RNA interference (Figure S5G-I). Immunofluorescence analysis revealed that MnSOD knockdown attenuated the ability of MnO₂-NPs to scavenge intracellular ROS (Figure 5H-I). These findings indicate that the antioxidant protective effect of MnO₂-NPs on hiPSC-EPCs depends on both the expression and functional activity of MnSOD.

### MnO_2_-NPs enhanced the resistance of hiPSC-EPCs to oxidative stress

**Figure 6:**
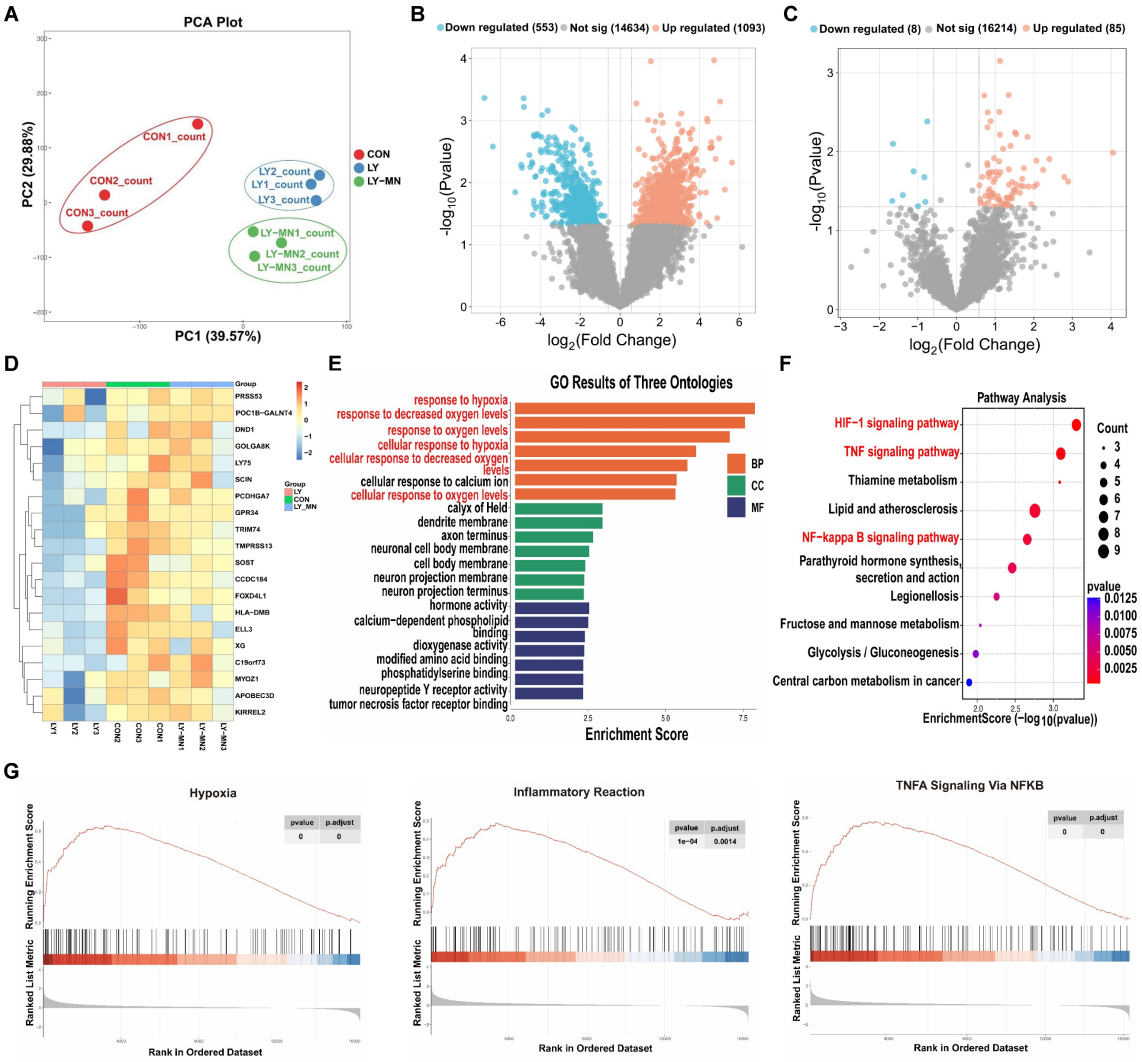
** Transcriptome sequencing of control, LY83583 and MnO_2_-NPs groups.** (**A**) PCA analysis. (**B**) Volcano diagrams of the control (CON) and LY83583 (LY). (**C**) Volcano diagrams of the MnO2-NPs (LY-MN) and LY. (**D**) Heatmap of DEGs among the three groups. (**E**) GO enrichment analysis between LY-MN and LY groups. (**F**) KEGG pathway enrichment analysis between LY-MN and LY groups. (**G**) GSE analysis of inflammatory response, TNFA signaling via NF-Κb, and hypoxia pathway between LY-MN and LY groups.

To further investigate the protective effect of the MnO_2_-NPs on hiPSC-EPCs under a high oxidative stress state, we performed RNA sequencing analysis on cells treated with either LY83583 or MnO_2_-NPs for 48 h. Principal components analysis (PCA) indicated the qualified heterogeneity of samples in every group (Figure 6A). Volcano plots demonstrated differences in gene expression in the Control (CON) and LY83583 (LY) groups (Figure 6B). A total of 16,307 gene were detected between the LY and MnO_2_-NPs (LY-MN) groups, including 85 up-regulated and 8 down-regulated differentially expressed genes (DEGs) (Figure 6C). Heat map showed the expression patterns of DEGs in the three groups (Figure 6D). Gene Ontology (GO) enrichment analysis revealed that DEGs were enriched in biological processes related to the response to hypoxia, response to decreased oxygen levels, cellular response to decreased oxygen levels, and other processes between the LY-MN and LY groups (Figure 6E). Encyclopedia of Genes and Genomes (KEGG) pathway enrichment analysis showed significant alterations in gene clusters associated with HIF-1, TNF, NF-κB, and other signaling pathways (Figure 6F). Furthermore, Western blot analysis showed that the levels of p-NF-κB and p-IκBα were increased following treatment with LY but decreased following treatment with LY-MN (Figure S5J-K). Gene set enrichment analysis (GSEA) analysis indicated that hypoxia, inflammatory response and TNFA signaling via NF-κB pathway were significantly enriched in the LY-MN group compared to the LY group (Figure 6G). These findings suggesting that enhanced cell survival, antioxidant capacity, and anti-inflammatory responses may underlie the protective effects of MnO_2_-NPs.

### HiPSC-EPCs incorporated with MnO_2_-NPs-Gel alleviated limb ischemia and promoted tissue repair

**Figure 7:**
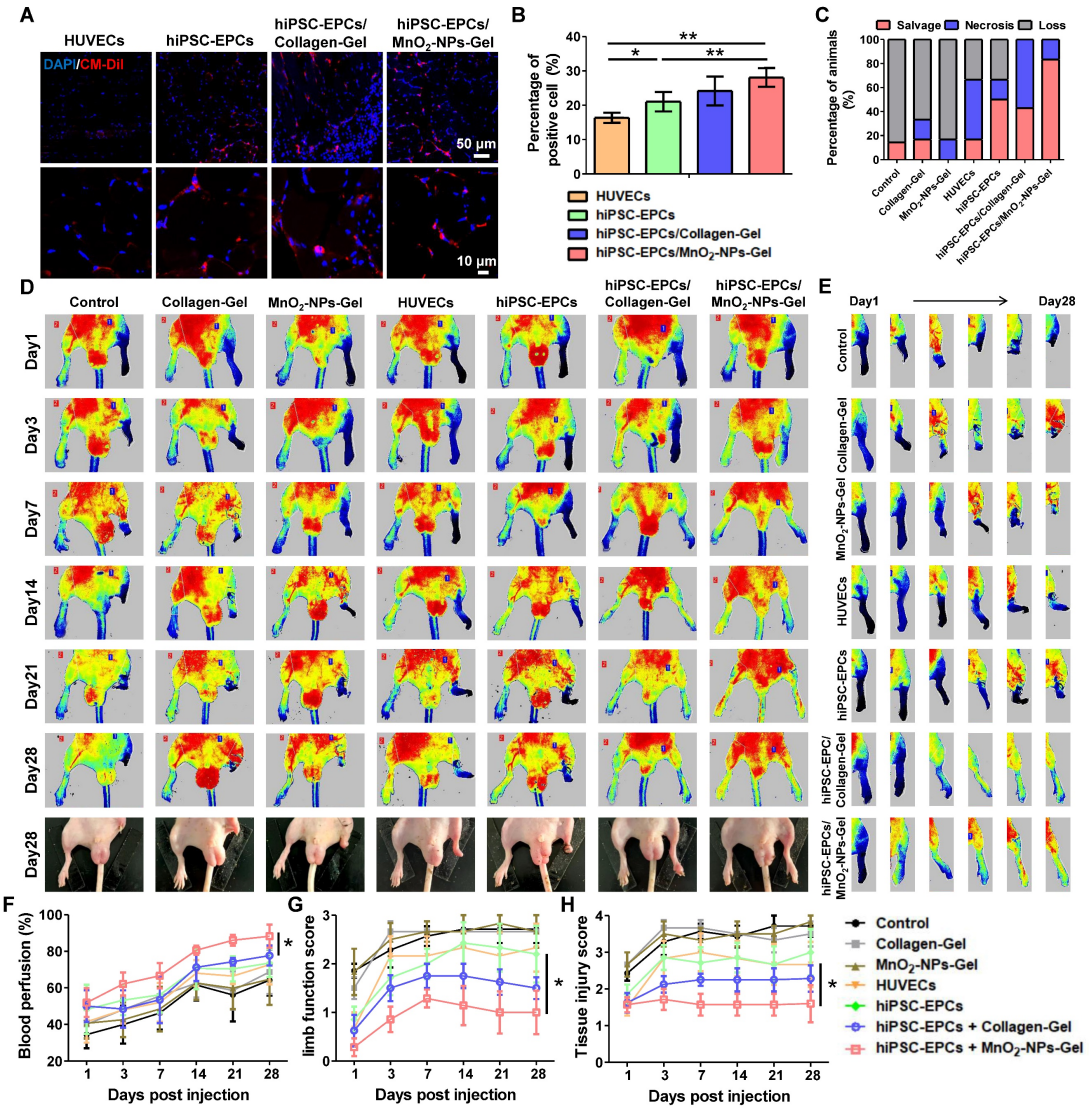
** MnO_2_-NPs-Gel extended the retention of hiPSC-EPCs in the ischemic tissues and promoted blood flow reconstruction.** (**A-B**) Representative images (**A**) and quantitative analysis (**B**) of viable cells in ischemic tissues of hindlimb ischemia mice treated with HUVECs, hiPSC-EPCs, Collagen-Gel/hiPSC-EPCs and MnO_2_-NPs-Gel/hiPSC-EPCs (*n* = 6). Top scale bar: 50 μm, bottom scale bar: 10 μm. (**C**) Quantitative analysis of the injury severity of hindlimb at day 28 post treatment (*n* = 6). (**D**) Representative laser doppler perfusion images of hindlimb ischemia mice treated with the Collagen-Gel, MnO_2_-NPs-Gel, HUVECs, hiPSC-EPCs, Collagen-Gel/hiPSC-EPCs or MnO_2_-NPs-Gel/hiPSC-EPCs. The bottom panel shows the recovery of injured hind limbs at 28 days post injection of each formula (*n* = 6). (**E**) Representative laser Doppler perfusion images of the right hindlimb of the mice treated with the treatments. (**F-H**) Quantitative analysis of the perfusion recovery (**F**), limb function score (**G**) and tissue damage score (**H**) of hindlimb ischemia mice (*n* = 6). The data represent mean ± *SD*. ns = no significance, *p < 0.05, **p < 0.01, ***p < 0.001, by one-way ANOVA or two-way ANOVA.

Following the validation of the protective effects of MnO_2_-NPs against oxidative stress, we examined whether the MnO_2_-NPs-Gel could exert its ROS-scavenging function to enhance the retention and survival of hiPSC-EPCs utilizing a mouse model of limb ischemia. Both the Collagen-Gel and MnO_2_-NPs-Gel enhanced the retention of implanted hiPSC-EPCs labeled with CM-Dil red fluorescent dye. In comparison to HUVECs, hiPSC-EPCs exhibited superior survival in ischemic lower limbs, attributable to their enhanced capacity to withstand oxidative stress (Figure 7A-B) 48. Furthermore, we performed immunofluorescence staining using a validated anti-human nuclear antigen antibody to assess the *in vivo* retention of hiPSC-EPCs in ischemic murine hind limb muscle tissue at 7 and 14 days post-transplantation. Human nuclear antigen-positive signals were detected in the ischemic muscle at day 7, whereas no specific signal was observed at day 14 (Figure S6A). Based on the encouraging results, we ultimately evaluated the potential of the MnO_2_-NPs-Gel in transporting hiPSC-EPCs *in vivo* for treating hindlimb ischemia. Injury severity results indicate that the MnO_2_-NPs-Gel/hiPSC-EPCs group has a more favorable therapeutic effect than the other groups. During the 28-day evaluation period, MnO_2_-NPs-Gel/hiPSC-EPCs group had the lowest incidence of foot necrosis (16.7%) and the highest limb preservation rate (83.3%) compared to those treated with other formulations (Figure 7C). To evaluate therapeutic efficacy, the perfusion status of the injured limbs was assessed. Without proangiogenic factors from therapeutic cells to facilitate angiogenesis, neither the Collagen-Gel and MnO_2_-NPs-Gel was able to attain the desired therapeutic effect. The presence of HUVECs or hiPSC-EPCs, which continuously secrete high concentration of proangiogenic factor, has also resulted in certain therapeutic effects when treated separately. In addition, Collagen-Gel increased the retention of hiPSC-EPCs in ischemic limbs, thereby further promoting the recovery of blood flow perfusion recovery. More importantly, MnO_2_-NPs-Gel improved hiPSC-EPC survival, resulted in optimal therapeutic outcomes (Figure 7D-F). Comparable tendencies can be observed in the results of limb function score and tissue injury score. In comparison with the Collagen-Gel/hiPSC-EPCs group, MnO_2_-NPs-Gel/hiPSC-EPCs group enhanced the repair of ischemic tissues, resulting in a significant reduction in both limb function score (Figure 7G) and tissue injury score (Figure 7H).

**Figure 8:**
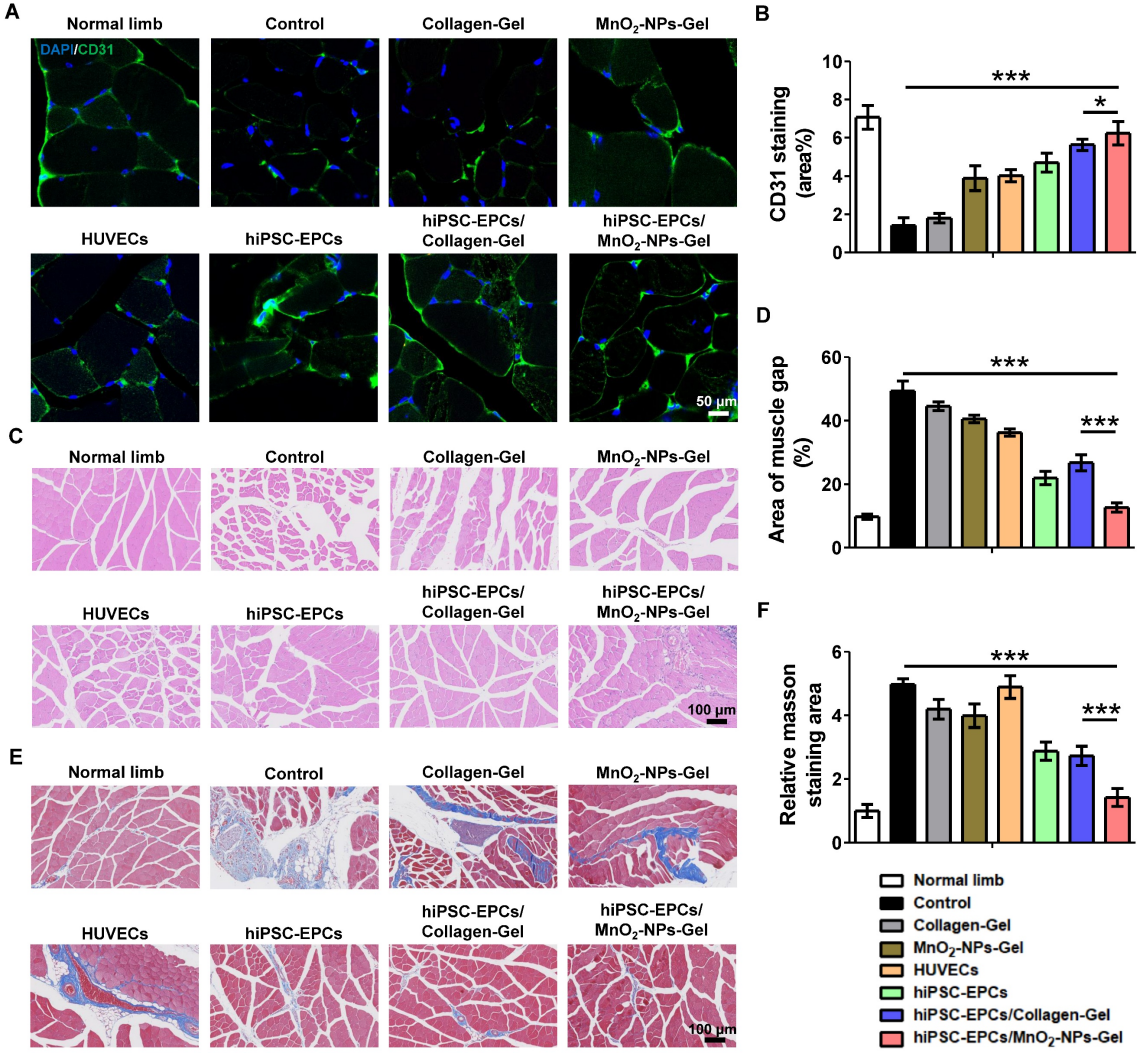
** HiPSC-EPCs improved tissue repair and ameliorated muscle fibrosis with the assistance of MnO_2_-NPs-Gel.** (**A-B**) Representative images (**A**) and quantitative analysis (**B**) of CD31 (green) staining in the ischemic tissues of hindlimb ischemia mice treated with the Collagen-Gel, MnO_2_-NPs-Gel, HUVECs, hiPSC-EPCs, Collagen-Gel/hiPSC-EPCs or MnO_2_-NPs-Gel/hiPSC-EPCs (*n* = 6). Top scale bar: 50 μm, bottom scale bar: 10 μm. (**C-D**) Representative images (**C**) and quantitative analysis (**D**) of HE staining in the ischemic tissues of hindlimb ischemia mice (*n* = 6). Scale bar: 100 μm. (**E-F**) Representative images (**E**) and quantitative analysis (**F**) of MASSON staining in the ischemic tissues of hindlimb ischemia mice (*n* = 6). Scale bar: 100 μm. The data represent mean ± *SD*. ns = no significance, *p < 0.05, ***p < 0.001, by one-way ANOVA or two-way ANOVA.

To investigate whether the superior therapeutic effects of MnO_2_-NPs-Gel/ hiPSC-EPCs are attributed to the enhanced proangiogenic effects, mice were euthanized on the 28th day after injection, and ischemic tissues were harvested. We found that the administration of MnO_2_-NPs-Gel/hiPSC-EPCs reduced tissue damage and no significant difference was observed in gastrocnemius muscle weight between ischemic and healthy limbs (Figure S6B-C). In comparison to the other groups, MnO_2_-NPs-Gel/hiPSC-EPCs group showed a greater staining of CD31 (green fluorescence), suggesting improved capillary formation in the injured limbs (Figure 8A-B). Moreover, the expression of *VEGFA* and *HGF* was significantly improved in the MnO₂-NPs-Gel/hiPSC-EPCs group under LY83583 compared to the Collagen-Gel/hiPSC-EPCs group (Figure S6D). Following the administration of MnO_2_-NPs Gel for hiPSC-EPCs delivery, increased capillary formation and improved blood perfusion resulted in enhanced tissue repair (Figure 8C-F).

## Discussion

CLI, the end-stage manifestation of peripheral arterial disease, causes severe pain, tissue ulceration, and frequent amputations, significantly compromising life quality of patients. Current clinical management primarily relies on pharmacotherapies (e.g., heparin, cilostazol) to inhibit platelet aggregation and preserve collateral vessel patency. However, these treatments fail to effectively restore blood perfusion to the affected limbs. Similarly, interventional or surgical revascularization is typically limited to large-caliber vessels and is ineffective for regenerating severely damaged microvasculature, with high risks of recurrence. Consequently, novel therapeutic strategies with enhanced efficacy for CLI are urgently needed. In this study, we developed an MnO_2_-NPs-Gel for targeted delivery of hiPSC-EPCs. This system uniquely overcomes a major limitation of conventional biomaterial strategies by not only scavenging extracellular ROS but also mitigating intracellular ROS generation within transplanted cells—a consequence of hypoxia-induced glycolysis that traditional approaches fail to address. Our findings demonstrate that the MnO_2_-NPs-Gel enhances hiPSC-EPCs retention, promotes the restoration of hindlimb blood flow, protects ischemic musculature from necrosis, and markedly improves limb salvage rates.

The pathogenesis of CLI is closely associated with vascular endothelial injury, which is recognized as a key mechanism underlying vascular dysfunction and impaired blood supply. ECs maintain vascular homeostasis, making their repair and regeneration a critical therapeutic effect for ischemic diseases. EPCs, as the precursors of ECs, exert potent pro-angiogenic effects primarily through the paracrine secretion of factors such as VEGF, HGF, IGF-1, PDGF, and FGF 54,55. However, endogenous EPCs in CLI patients are often depleted and functionally impaired, rendering them unsuitable for therapeutic applications 56, 57. We therefore employed hiPSC-EPCs, selected for their superior proliferative capacity, robust paracrine activity, and notably, enhanced intrinsic antioxidant resistance compared to HUVECs 58. Consistent with this, hiPSC-EPCs demonstrated higher basal expression of MnSOD than HUVECs. While our initial studies showed marginally better therapeutic outcomes compared to HUVECs in CLI models, attributed to improved retention and pro-angiogenic function, the efficacy remained suboptimal due to the hostile ischemic microenvironment.

A defining feature of the ischemic limb is the massive accumulation of ROS, which induces catastrophic oxidative damage through two converging pathways. Exogenous ROS originating from the extracellular hypoxic and inflammatory microenvironment cause membrane lipid peroxidation and calcium dyshomeostasis, triggering apoptosis. Endogenous ROS generated intracellularly, primarily through mitochondrial dysfunction and exacerbated by hypoxia-induced glycolytic shifts, directly damage cellular lipids, proteins, and DNA, leading to organelle failure and cell death 59-62. Conventional biomaterials, while capable of scavenging extracellular ROS, offer no protection against this detrimental intracellular ROS surge.

Our study elucidates a breakthrough cytoprotective mechanism conferred by MnO_2_-NPs within the hydrogel: a dual-action strategy against oxidative stress. Firstly, the MnO_2_-NPs act catalytically within the extracellular space, decomposing harmful ROS species (H_2_O_2_, O_2_^-^, ·OH) into oxygen 31, 63-65, thereby alleviating exogenous oxidative stress and simultaneously ameliorating local hypoxia. The TMB oxidation assay and the peroxide detection assay both confirmed the catalytic decomposition of H₂O₂ by MnO₂-NPs. Second, and most significantly, Mn^2+^ released during the biodegradation of MnO_2_-NPs is internalized by adjacent cells via specific transporters (e.g., Smf1/Smf2) and delivered to the mitochondria 66. Within the mitochondrial matrix, Mn^2+^ serves as an essential cofactor for the biosynthesis and activation of MnSOD. This enzymatic upregulation promotes the intrinsic capacity of hiPSC-EPCs to neutralize mitochondrially-derived endogenous superoxide radicals (O_2_^-^), thereby mitigating intracellular oxidative damage. Direct experimental evidence supporting this was provided by DHE staining, which revealed a significant decrease in intracellular superoxide levels in hiPSC-EPCs treated with MnO_2_-NPs compared to untreated controls. Furthermore, knockdown of MnSOD abolished the protective effect. This dual extracellular scavenging and intracellular enzymatic defense mechanism represents a substantial advancement over prior biomaterial-based delivery systems. It is worth noting that nanozymes based on iron, manganese, titanium, and copper have demonstrated excellent ROS-scavenging activity in myocardial infarction, acute lung injury, chronic wounds, and tumor therapy 67-71. Moreover, in preclinical models, both bioengineered mesenchymal stem cells and certain antioxidant hydrogels have shown potent therapeutic effects 72, 73.

While theoretical concerns exist regarding Mn^2+^ potentially reacting with residual H_2_O_2_ to generate harmful hydroxyl radicals (·OH) via Fenton-like reactions, this process is critically dependent on non-physiological conditions (pH < 5.0, photoirradiation, ultrasound, or high glutathione) 74, 75 that are irrelevant to the ischemic limb microenvironment. Our data demonstrating net ROS reduction strongly suggest that MnO_2_-NPs effectively prevent ·OH accumulation by rapidly depleting its precursor, H_2_O_2_. It is important to note that the biosafety of manganese-based nanomaterials has been systematically evaluated in our previous study and others 29, 76. Additionally, the collagen hydrogel component synergistically contributes to therapeutic efficacy. As a major extracellular matrix protein, collagen provides excellent biocompatibility, low immunogenicity, and crucially, contains cell-adhesive motifs that promote hiPSC-EPCs adhesion, survival, and proliferation—advantages over materials like alginate which lack such binding sites 77, 78. The hydrogel enables controlled release of Mn^2+^, mitigating potential toxicity 29, 30. Furthermore, the gelation time determines the balance between injectability and *in situ* stabilization: a sufficiently rapid gelation after administration is beneficial for preventing material dispersion and cell leakage, while avoiding excessively fast solidification that could impair homogeneous cell encapsulation or delivery. Meanwhile, the mechanical properties of the hydrogel, especially stiffness and viscoelasticity, are known to influence cell retention by resisting deformation from surrounding tissue motion and fluid flow, while simultaneously providing a permissive microenvironment for cell survival and migration 79, 80. The degradation of collagen hydrogel *in vivo* is governed by enzymatic cleavage (e.g., collagenase and matrix metalloproteinase) and gradual hydrolysis under physiological conditions. It undergoes gradual biodegradation *in vivo* over several weeks, providing sufficient structural support during the early healing phase before being resorbed 81.

In this study, although BALB/c nude mice can effectively avoid immune rejection of hiPSC-EPCs, their fundamental limitation lies in the lack of a fully functional immune system, particularly the absence of mature T lymphocytes. This defect makes the model incapable of accurately simulating the dynamic, multilayered immune regulatory processes intrinsic to human peripheral ischemia and repair.

## Conclusions

**Figure 9:**
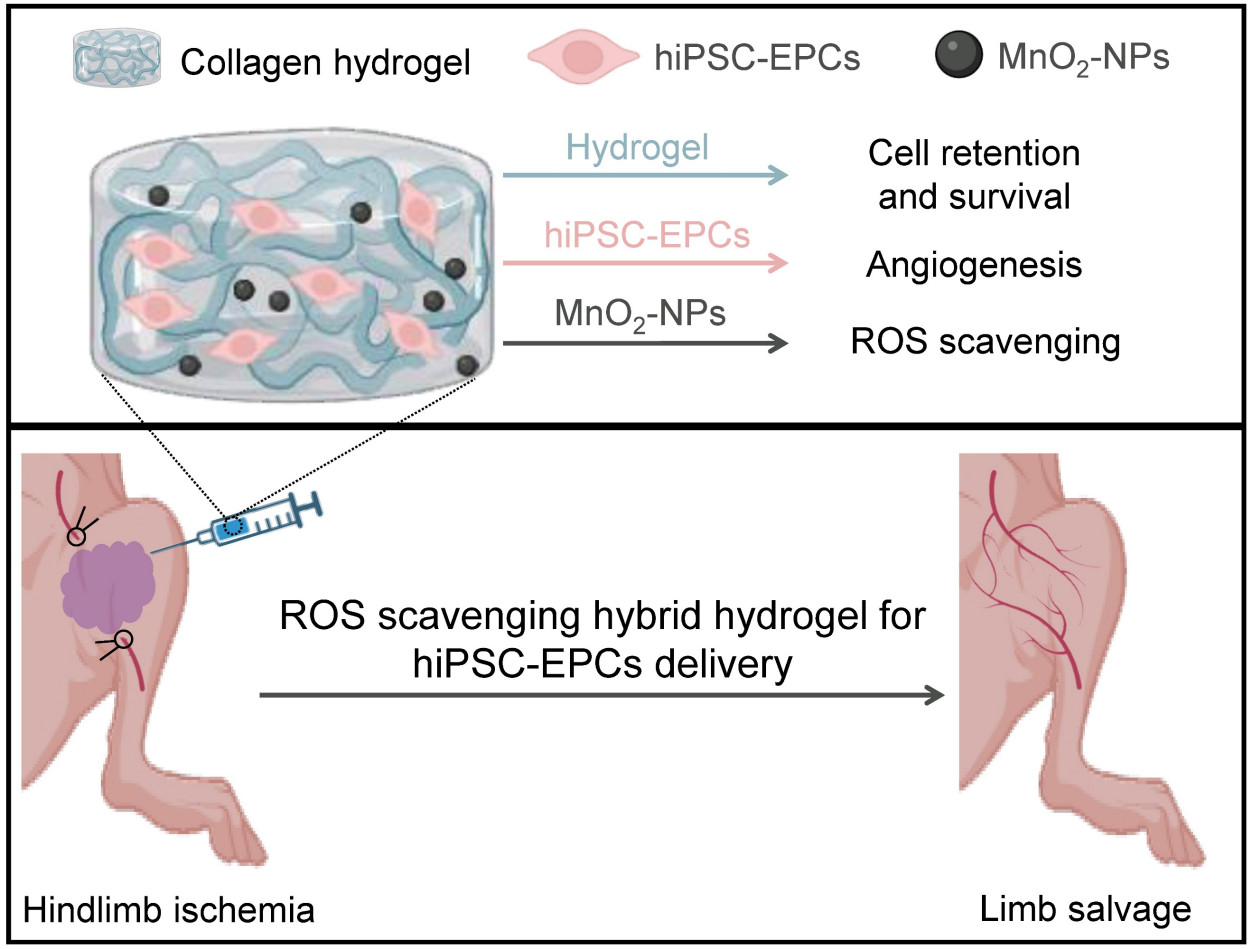
** Schematic illustration of the collagen/MnO_2_-NPs hybrid hydrogel with ROS scavenging ability for hiPSC-EPCs delivery and limb ischemia therapy.** After the encapsulation of hiPSC-EPCs and then intramuscular injection into the ischemic limbs of mice, the thermal-triggered *in situ* gelation of collagen could maintain hiPSC-EPCs at the injection site and the MnO_2_-NPs entrapped into the hydrogel framework could scavenge the high level of ROS in the ischemic tissues to promote the stem cell survival. Subsequently, hiPSC-EPCs could enhance angiogenesis, leading to increased blood perfusion in the ischemic limbs and superior limb salvage.

In conclusion, the MnO_2_-NPs-Gel system establishes a new paradigm for cell therapy in CLI (Figure 9). By ingeniously combining extracellular ROS scavenging and oxygen generation with the targeted intracellular upregulation of the key antioxidant enzyme MnSOD in transplanted hiPSC-EPCs, it creates a profoundly protective microenvironment. This dual-action strategy overcomes the critical limitation of intracellular ROS-mediated damage that plagues conventional approaches, thereby maximizing hiPSC-EPCs survival, paracrine function, and ultimately, therapeutic revascularization and limb salvage.

## Supplementary Material

Supplementary figures (S1-S6). Supplementary tables S1 and S2.

## Data Availability

All data supporting the findings of this study are available from the corresponding author upon reasonable request.

## Abbreviations

CLI: critical limb ischemia
EPCs: Endothelial progenitor cells
hiPSC: Human induced pluripotent stem cell
hiPSC-EPCs: Human-induced pluripotent stem cell-derived endothelial progenitor cells
ROS: Reactive oxygen species
MnO_2_-NPs: Manganese dioxide nanoparticles
HUVECs: Human umbilical vein endothelial cells MMP: Mitochondrial membrane potential
MnSOD: Manganese superoxide dismutase
CuZnSOD: Copper-zinc-superoxide dismutase
VEGF: Vascular endothelial growth factor
VEGFR: Vascular endothelial growth factor receptor
CD31: Platelet and endothelial cell adhesion molecule 1
CD34: CD34 molecule
CD144: cadherin 5
SOX2: SRY-box transcription factor 2
MnSOD: Superoxide dismutase 2
CuZnSOD: superoxide dismutase 1
OCT4: POU class 5 homeobox 1
