# Supplementary material for: Dual ROS modulation by MnO2-integrated collagen hydrogel enhances hiPSC-derived endothelial progenitor cell therapy for critical limb ischemia
Source: Theranostics. 2026 Apr 22;16(11):6164–83. doi: 10.7150/thno.127711 (PMC13142243; doi:10.7150/thno.127711)
Supplement: Supplementary file 1 — Supplementary figures (S1-S6). Supplementary tables S1 and S2. [file thnov16p6164s1.pdf]

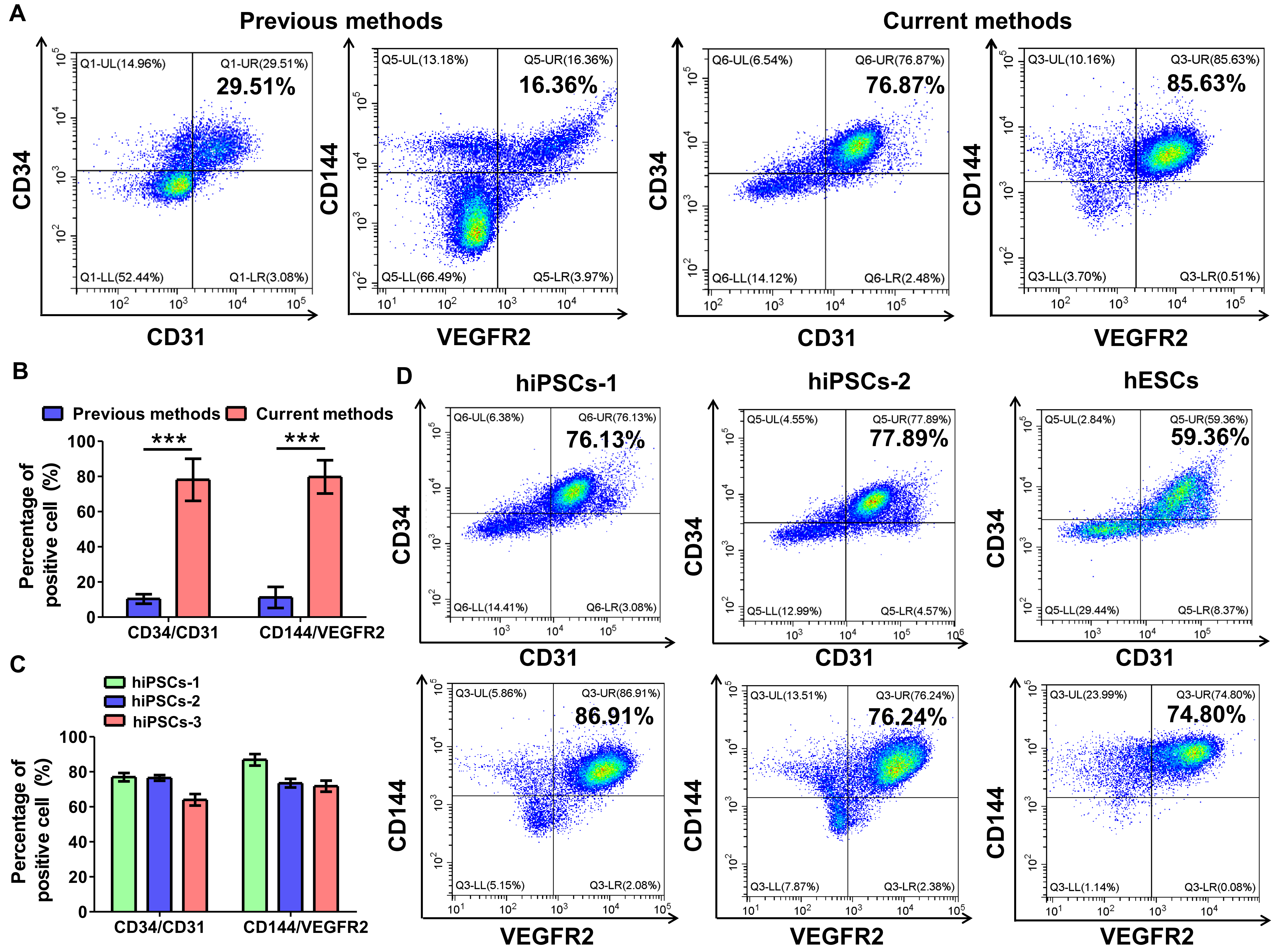

Figure S1. Differentiation of different human induced pluripotent stem cell lines into EPCs. (**A**) Co-expression of CD34/CD31 or CD144/VEGFR2 for hiPSC-EPCs was detected by FACS. (**B**) Quantitative analysis of the $CD34^+/CD31^+$ or $CD144^+/VEGFR2^+$ hiPSC-EPCs (n = 3). (**C**) Quantitative analysis of the $CD34^+/CD31^+$ or $CD144^+/VEGFR2^+$ EPCs (n = 3). (**D**) Co-expression of CD34/CD31 or CD144/VEGFR2 for hUiPSC-EPCs was detected by FACS. The data represent mean ± *SD*. ***$p < 0.001$, by one-way ANOVA or Student's *t* test.

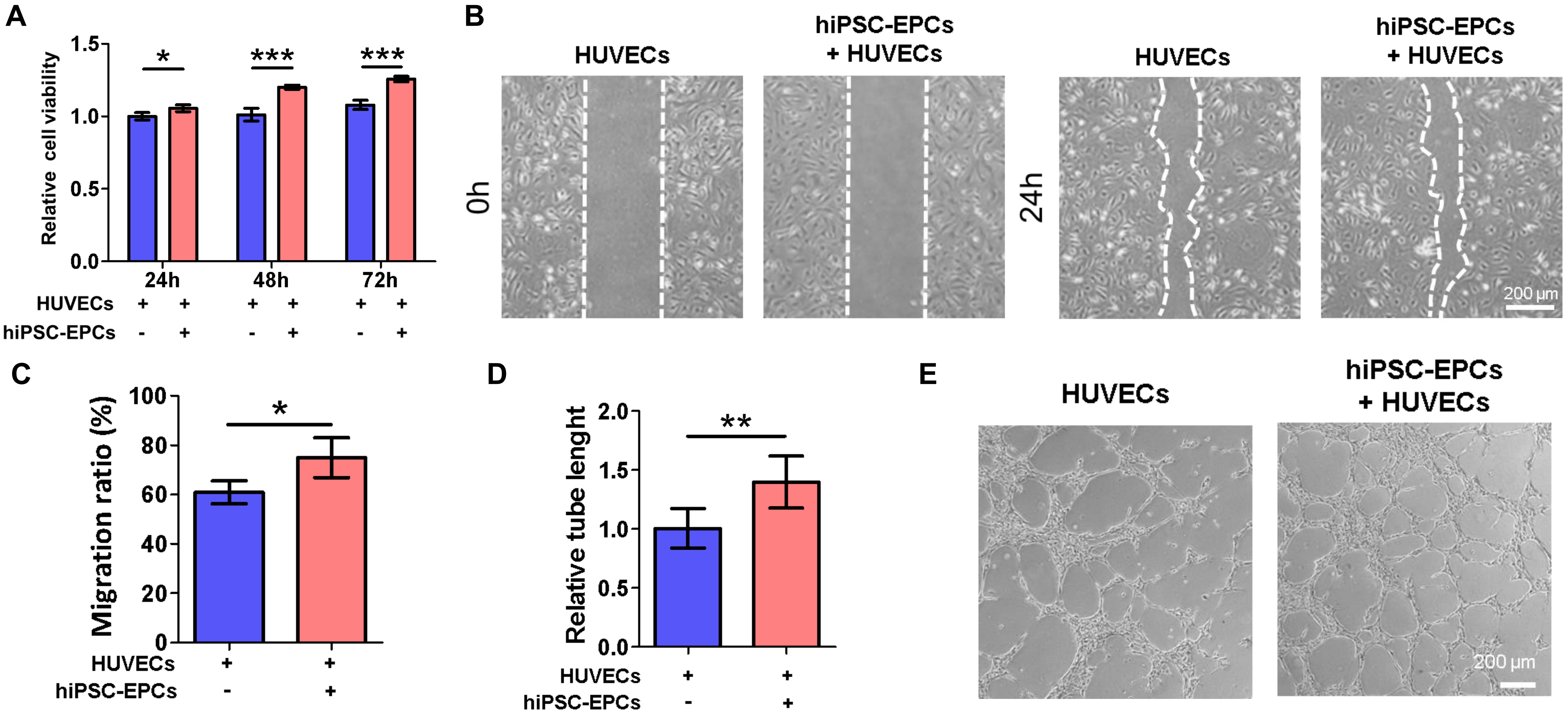

Figure S2. HiPSC-EPCs promote the proliferation, migration, and tube formation of HUVECs in vitro. (**A**) Cell proliferation of HUVECs after co-culture with hiPSC-EPCs was analyzed using a CCK-8 kit (n = 3). (**B-C**) Representative images (**B**) and migration ratio (**C**) of HUVECs after co-culture with hiPSC-EPCs in wound healing assay (n = 3). (**D-E**) relative tube legnght (**D**) and representative images (**E**) and of HUVECs after co-culture with hiPSC-EPCs in tube formation assay (n = 3). The data represent mean ± *SD*. ns = no significance, *p < 0.05, **p < 0.01, ***p < 0.001, by one-way ANOVA or Student's t test.

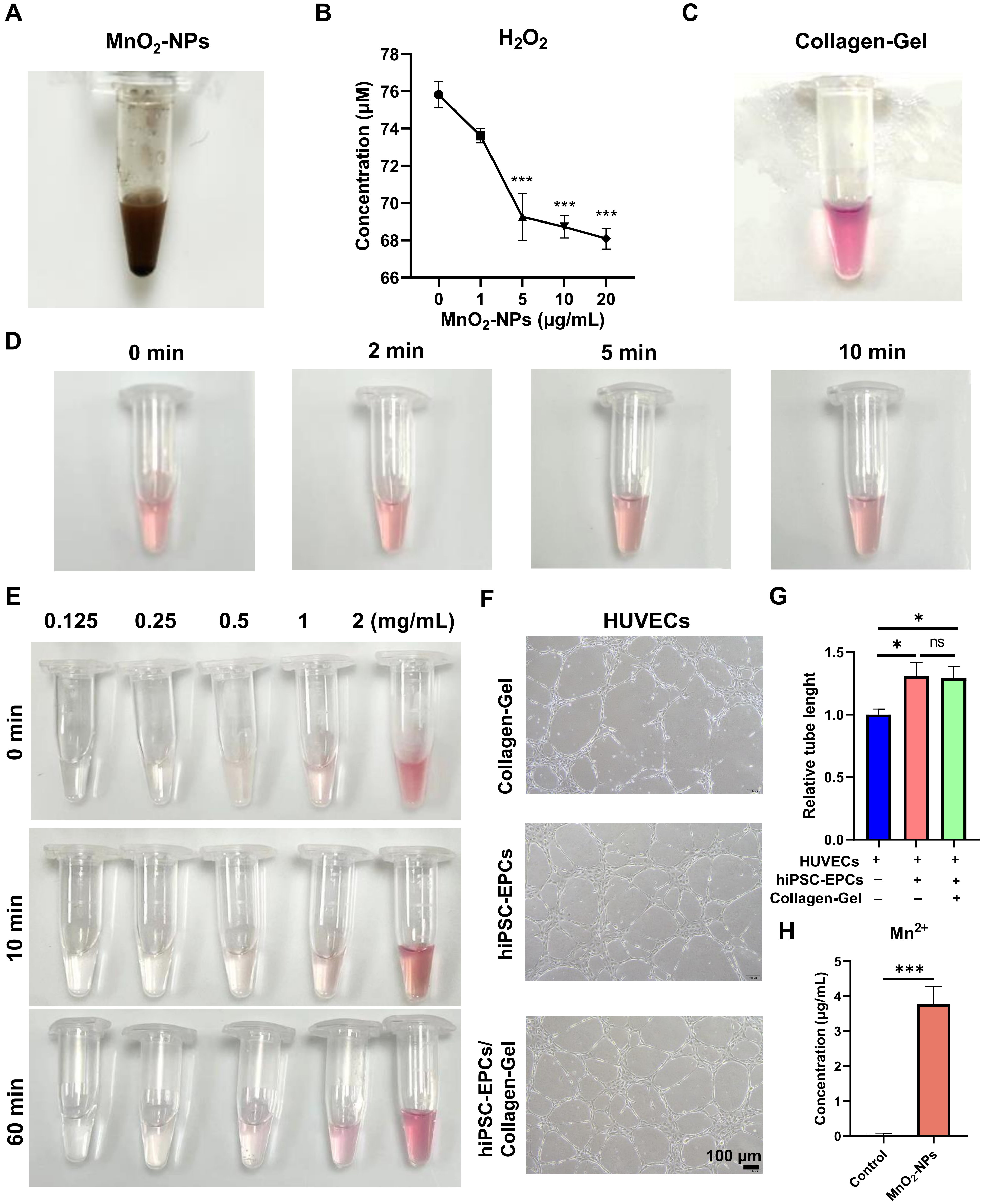

Figure S3. The properties and of MnO2-NPs and Collagen-Gel. (**A**) Representative images of MnO2-NPs. (**B**) H_2O_2 decomposition catalyzed by MnO2-NPs was quantified using a commercial peroxide assay kit (n = 3). (**C**) Representative images of Collagen-Gel. (**D**) Formation time of 2 mg/mL Collagen-Gel solution to complete the gelation. (**E**) Critical concentration of Collagen-Gel for gelation. (**F**) Representative images of HUVECs after co-culture with hiPSC-EPCs and hiPSC-EPCs/Collagen-Gel in tube formation assay (n = 3). (**G**) Relative tube legnght of HUVECs. (**H**) Inductively coupled plasma mass spectrometry (ICP-MS) quantified intracellular Mn^{2+} levels in hiPSC-EPCs following 48-hour incubation with MnO-NPs (n = 3). The data represent mean ± SD. ***$p < 0.001$, by one-way ANOVA or Student's t test.

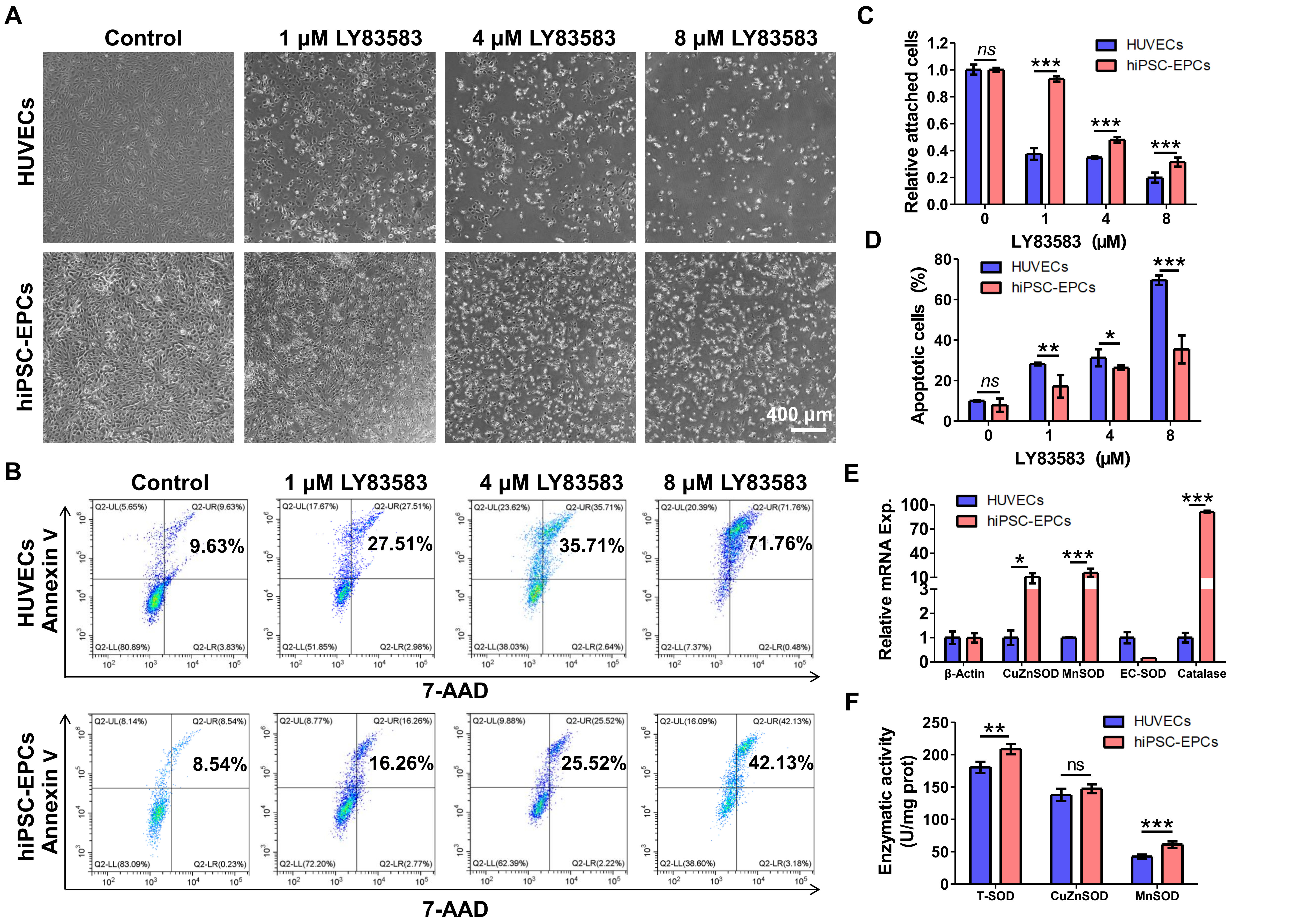

Figure S4. HiPSC-EPCs had stronger resistance to oxidative stress–induced apoptosis as compared with HUVECs. (**A**) Morphological characteristics of hiPSC-EPCs and HUVECs incubated with 4 μM LY83583 for 48 hours. Scale bar: 400 μm. (**B**) Apoptotic hiPSC-EPCs and HUVECs incubated with 4 μM LY83583 for 48 h were detected by annexin-V and 7-AAD staining using FACS. (**C**) Comparison of attached cells of hiPSC-EPCs and HUVECs (*n* = 3). (**D**) Comparison of apoptotic cells of hiPSC-EPCs and HUVECs (*n* = 3). (**E**) Comparison of mRNA levels of CuZnSOD, MnSOD, EC-SOD and catalase in hiPSC-EPCs and HUVECs (*n* = 3). (**F**) Comparison of enzymatic activity of total SOD, CuZnSOD and MnSOD in hiPSC-EPCs and HUVECs (*n* = 3). The data represent mean ± *SD*. ns = no significance, *$p < 0.05$, **$p < 0.01$, ***$p < 0.001$, by two-way ANOVA.

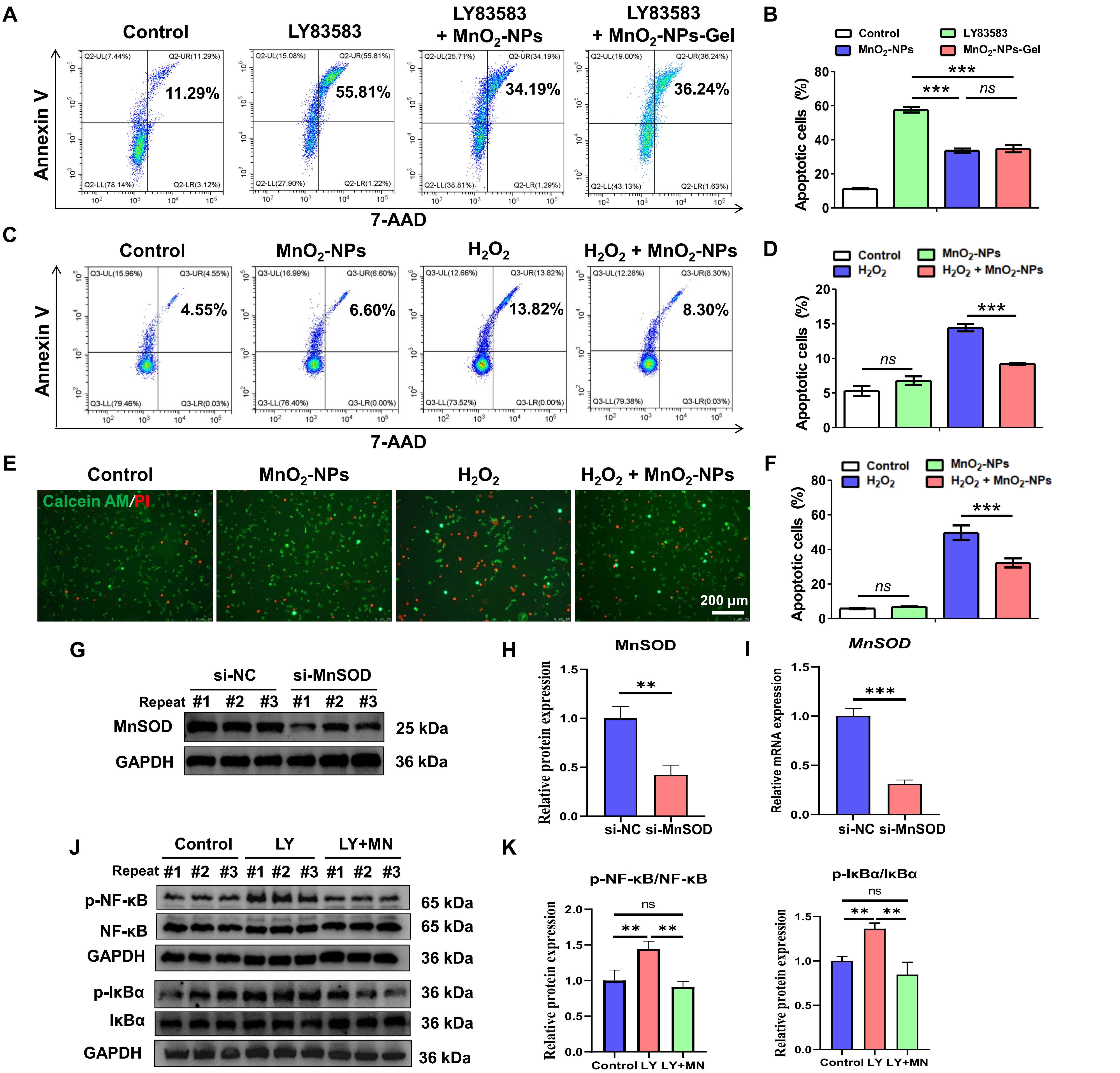

Figure S5. The capacity of MnO_2-NPs to enhance cell survival in oxidative stress. (**A**) Apoptotic hiPSC-EPCs incubated with 4 μM LY83583 for 48 h were detected by annexin-V and 7-AAD staining using FACS. (**B**) Quantitative analysis of apoptotic hiPSC-EPCs (n = 3). (**C-D**) Apoptotic cells (**C**) and quantitative analysis (**D**) of hiPSC-EPCs incubated with 100 μM H_2O_2 for 48 h were detected by annexin-V and 7-AAD staining using FACS (n = 3). (**E-F**) Representative images (**E**) and quantitative analysis (**F**) of live-dead assay of hiPSC-EPCs incubated with 100 μM H_2O_2 for 48 h (n = 3). (**G-H**) Western blot analysis of MnSOD (n = 3). Repeat #1, #2, and #3 represent three independent biological replicates. (**I**) The knockdown efficiency of *MnSOD* was assessed by q-PCR (n = 3). (**J-K**) Western blot analysis of p-NF-κB, NF-κb, p-IκBα and IκBα following treatment with LY83583 (LY) or LY83583/MnO_2-NPs (LY+MN) for 48 h (n = 3).). Repeat #1, #2, and #3 represent three independent biological replicates. The data represent mean ± *SD*. *ns* = no significance, $^{**}p < 0.01$, $^{***}p < 0.001$, by one-way ANOVA or student's t test.

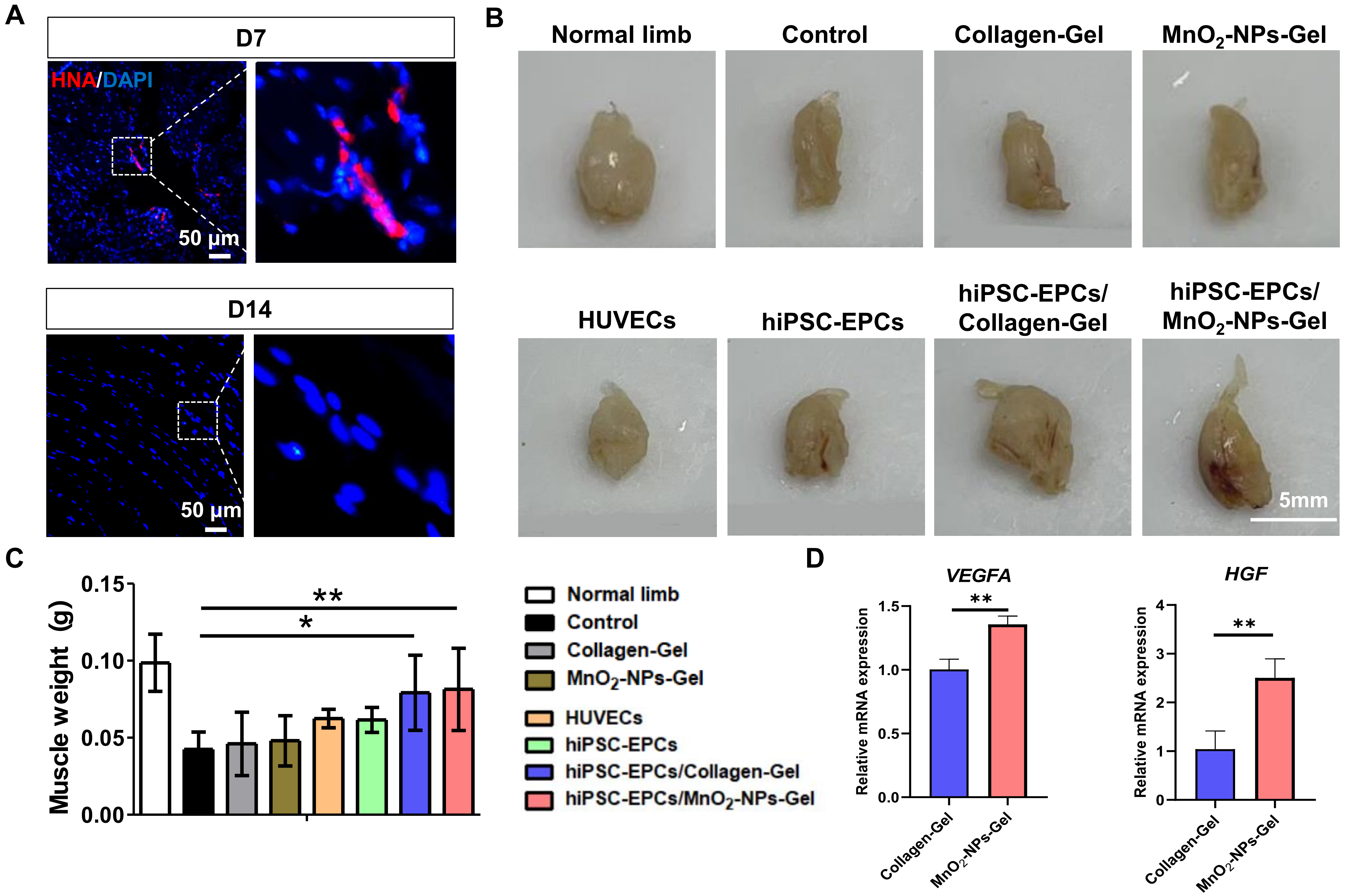

Figure S6. The capacity of hiPSC-EPCs with MnO_2-NPs-Gel to protect musle from ischemic injury. (**A**) Immunofluorescence staining analysis of the in vivo retention of hiPSC-EPCs in ischemic murine hind limb muscle tissue at 7 and 14 days post-transplantation (n = 3). HNA, human nuclear antibody. (**B**) Representative morphology of the gastrocnemius muscle of hindlimb ischemia mice treated withthe Collagen-Gel, MnO_2-NPs-Gel, HUVECs, hiPSC-EPCs, Collagen-Gel/hiPSC-EPCs or MnO_2-NPs-Gel/hiPSC-EPCs (n = 6). (**C**) Quantitative analysis of muscle weight of the ischemic tissues of hindlimb ischemia mice. (**D**) The expression of *VEGFA* and *HGF* was detected by q-PCR in the MnO_2-NPs-Gel/hiPSC-EPCs group and the Collagen-Gel/hiPSC-EPCs group under LY83583 treatment (n = 3). The data represent mean ± *SD*. *$p < 0.05$, **$p < 0.01$, by one-way ANOVA or Student's t test.

Table S1. Antibodies information

Antibodies	Company	Catalog#
FITC-anti-human-CD34	Biolegend	343604
APC/Cyanine7-anti-human-CD31	Biolegend	303120
PerCP/Cyanine5.5-anti-human-CD144	Biolegend	348510
APC-anti-human-CD309(VEGFR2)	Biolegend	359916
Anti-human-SOD1	Affinity Biosciences	AF5198-100
Anti-human-SOD2(MnSOD)	Affinity Biosciences	AF5144-100
Anti-human-Catalase	Affinity Biosciences	DF7545-100
Anti-human-β-actin	SAB	52901
Anti-rabbit IgG HRP-linked antibody	Cell SignalingTechnology	7074P2
Anti-mouse IgG HRP-linked antibody	Cell SignalingTechnology	7076P2
Anti-human-SOX2	Abcam	ab97959
Anti-human-Oct4	Abcam	ab184665
Anti-human-CD34	Abcam	ab81289
Anti-human-VEGFR2	Abcam	ab39378
Anti-Nuclei Antibody	Merck	MAB1281
Anti-NF-κB	Abways	CY5034
Anti-p-NF-κB	Abways	CY6372
Anti-IκBα	Abways	CY5026
Anti-p-IκBα	Abways	CY6280
Anti-GAPDH	Affinity Biosciences	T0004

Table S2. Sequences of primers

Primer name		Sequence
β-actin	forward primer	CATGTACGTTGCTATCCAGGC
	reserved primer	CTCCTTAATGTCACGCACGAT
CuZnSOD	forward primer	AATAAGTGCCATACAGGGTT
	reserved primer	AAAGGTGGAAATGAAGAAAG
MnSOD	forward primer	GGAAGCCATCAAACGTGACTT
	reserved primer	CCCGTTCCTTATTGAAACCAAGC
ECSOD	forward primer	ATGCTGGCGCTACTGTGTTC
	reserved primer	CTCCGCCGAGTCAGAGTTG
Catalase	forward primer	TGTTGCTGGAGAATCGGGTTC
	reserved primer	TCCCAGTTACCATCTTCTGTGTA
Nanog	forward primer	TTTGTGGGCCTGAAGAAAACT
	reserved primer	AGGGCTGTCCTGAATAAGCAG
Oct4	forward primer	CTGGGTTGATCCTCGGACCT
	reserved primer	CCATCGGAGTTGCTCTCCA
SOX2	forward primer	GCCGAGTGGAAACTTTTGTCG
	reserved primer	GGCAGCGTGTACTTATCCTTCT
CD31	forward primer	CCAAGGTGGGATCGTGAGG
	reserved primer	TCGGAAGGATAAAACGCGGTC
VEGFR2	forward primer	CCTGTATGGAGGAGGAGGAA
	reserved primer	CGGCTCTTTCGCTTACTGTT
CD34	forward primer	TTTGCTTGCTGAGTTTGCTG
	reserved primer	ATTTGAAAATGTTCCCTGGGT
CD144	forward primer	CATCTTCCCAGGAGGAACAG
	reserved primer	AGAGCTCCACTCACGCTCAG
GAPDH	forward primer	GTCTCCTCTGACTTCAACAGCG
	reserved primer	ACCACCCTGTTGCTGTAGCCAA
VEGFA	forward primer	AAGCGCAAGAAATCCCGGTA
	reserved primer	CGCGAGTCTGTGTTTTTGCA
HGF	forward primer	ACCCTGGTGTTTCACAAGCA
	reserved primer	TGATCCCAGCGCTGACAAAT
